# LILRB4 regulates circadian disruption-induced mammary tumorigenesis via non-canonical WNT signaling pathway

**DOI:** 10.1038/s41388-025-03597-5

**Published:** 2025-10-16

**Authors:** Olajumoke Ogunlusi, Mrinmoy Sarkar, Kayla Carter, Arhit Chakrabarti, Devon J. Boland, Tristan Nguyen, James Sampson, Christian Nguyen, Danielle Fails, Yava Jones-Hall, Loning Fu, Gus Wright, Da Mi Kim, James J. Cai, Bani Mallick, Alex C. Keene, Jeff R. Jones, Tapasree Roy Sarkar

**Affiliations:** 1 Department of Biology, Texas A&M University, College Station, TX USA; 2 Department of Statistics, Texas A&M University, College Station, TX USA; 3 Texas A&M Institute of Genome Sciences & Society (TIGSS), College Station, TX USA; 4 Department of Biomedical Engineering, Texas A&M University, College Station, TX USA; 5 Fortis Life Sciences, Montgomery, TX 77356 USA; 6 Veterinary Pathobiology, Texas A&M University, College Station, TX USA; 7 Department of Medicine/Molecular Cell Biology, Baylor College of Medicine, Houston, TX USA; 8 Flow Cytometry Facility, Veterinary Pathobiology, Texas A&M University, College Station, TX USA; 9 Veterinary Integrative Biosciences, Texas A&M University, College Station, TX USA; 10 Texas A&M Center for Biological Clocks Research, College Station, TX USA

**Keywords:** Breast cancer, Biomarkers

## Abstract

Epidemiological studies have shown that circadian rhythm disruption (CRD) is associated with the risk of breast cancer. However, the role of CRD in mammary gland morphology and aggressive basal mammary tumorigenesis and the molecular mechanism underlying CRD-induced carcinogenesis remain unknown. To investigate the effect of CRD on aggressive tumorigenesis, a genetically engineered mouse model of aggressive breast cancer was used. The impact of CRD on the tumor microenvironment was investigated using the tumors from LD12:12 and CRD mice via scRNA-seq, flow cytometry, multiplexing immunostaining, and realtime PCR. The effect of LILRB4-immunotherapy on CRD-induced tumorigenesis was also investigated. Here we investigated and identified the impact of CRD on basal tumorigenesis and mammary gland morphology. We found that chronic CRD disrupted mammary gland morphology, increased lung metastasis, and induced an immunosuppressive tumor microenvironment by enhancing LILRB4 expression. Furthermore, targeted immunotherapy against LILRB4 reduced CRD-induced immunosuppressive microenvironment and lung metastasis. Finally, we showed that LILRB4 regulates CRD-induced mammary tumorigenesis via a non-canonical WNT signaling pathway. These findings identify and implicate LILRB4 as a link between CRD and aggressive mammary tumorigenesis and establish the potential role of the targeted LILRB4a immunotherapy as an inhibitor of CRD-induced lung metastasis.

## Background

The circadian clock regulates the expression of several genes that impact physiology, metabolic processes, and health outcomes [1–3]. Epidemiological studies have reported that circadian rhythm disruption (CRD), such as that which occurs during shift work or travel across time zones, affects human health and increases the risk of developing cancer, metabolic disorders, and cardiovascular disease [4]. According to the National Health Interview Survey, 12–35% of the US population works irregular schedules, including night and rotating shifts [5]. CRD increases the risk of different types of cancer, including lung [6], colon [7], and breast [8] cancers.

Shiftwork was found to increase the incidence of breast cancer in nurses by approximately 50%, suggesting a critical role for the circadian clock in breast cancer pathogenesis [9]. Circadian clocks regulate the rhythmic expression of numerous genes in breast tissues [10]. Triple-negative breast cancer (TNBC) or basal phenotype [11] encompasses a breast tumor subtype that is clinically negative for the expression of the estrogen (ER) and progesterone (PR) receptors and lacks overexpression of the Human Epidermal Growth Factor Receptor 2 (HER2) protein [12]. TNBC is responsible for more than 15–20% of all breast cancers, and it is very aggressive, with a mortality rate of 40% [13]. However, the effect of CRD on aggressive TNBC is not yet known, and the precise mechanisms, underlying CRD-induced tumorigenesis, have not been studied yet.

The mammalian circadian machinery consists of an autoregulatory transcription-translation feedback loop, where the “positive elements” circadian locomotor output cycle kaput (CLOCK) and brain and muscle aryl hydrocarbon receptor nuclear translocator (ARNT)-like protein 1 (BMAL1) heterodimerize through their PAS domains and activate the transcription of the “negative elements,” i.e., the period genes (*PER1* and *PER2)* and the cryptochrome genes (*CRY1* and *CRY2)*. The PER/CRY heterodimers inhibit the transcription of their gene(s) by blocking CLOCK/BMAL1-dependent transactivation [1]. The expression of core circadian clock genes is frequently dysregulated in human tumors, indicating the tumor-suppressive role of the molecular clock [7, 14–17]. Epidemiological studies have also emphasized that the risk of developing cancer increases with increasing years of shift work [9], indicating that longitudinal experiments are better suited for characterizing how CRD may affect tumorigenesis.

Here, we investigated how the loss of circadian function impacts the mammary gland, tumorigenesis, and the tumor microenvironment (TME). We specifically examined the role of leukocyte immunoglobulin-like receptor 4a (LILRB4a or LILRB4), which is known to suppress immunity in acute myeloid leukemia (AML) and solid tumors [18]. Additionally, we investigated whether the elevation of LILRB4 expression could be a key molecular event in CRD-induced mammary tumorigenesis and immunosuppressive TME. Overall, this study shows how circadian desynchronization affects mammary gland morphology and aggressive mammary tumorigenesis, identifies a compelling target for immunotherapy, and identifies the signaling pathway of CRD-induced tumorigenesis.

## Methods

### Animal husbandry

The mice were housed under 12 h light and 12 h dark (LD 12:12) and CRD conditions at room temperature (25 °C), and food and water were provided *ad libitum*. The GEMM i.e., FVB-Tg(C3-1-Tag)cJeg/JegJ mice (Jax #013591), were caged until they were 27 weeks old. Normal FVB mice (Jax #001800) (6-weeks old), and BALB/cJ mice (Jax #000651) were also used in this study. All animal care and treatments were in accordance with the Texas A&M University Animal Care and Use Committee under protocol #2022-0094. Mice were grouped randomly for exposure to standard light conditions (LD 12:12) or CRD light conditions (consisting of an 8-h light phase advance repeated every two days) [6, 19, 20]. Zeitgeber time (ZT) 0 corresponded to the onset of light, while ZT12 corresponded to the onset of dark. The LD 12:12 and CRD animal groups were provided access to running wheels (Columbus Instruments) for three weeks. ActogramJ (ImageJ) was used to assess voluntary running activities [21].

### Real-time PCR

Total RNA was extracted from mammary glands or tumors using Quick-RNA™ MiniPrep (Zymo Research), and the purity was analyzed by DeNovix DS-11 Series nanodrop Spectrophotometer (DeNovix). RNA from each sample was reverse transcribed to cDNA by High-Capacity cDNA Reverse Transcription Kit (Applied Biosystems). Analysis for gene expression by quantitative real-time PCR was performed using SSO Advanced Universal SYBR Green Supermix (BioRad). We used *Gapdh* and *Rplp0 (U36b4)* [19] as housekeeping genes.

### Human CRD analysis for breast cancer patients

Bulk RNA-sequencing data from the publicly available TCGA BRCA dataset was used to explore the expression pattern of the core circadian genes in humans. The TCGA dataset was downloaded from the UCSC Xena database [22]. The repository for the code used in the analysis can be found here (https://github.com/Arhit-Chakrabarti/TCGA-BRCA-Analysis). The patients were segregated according to the analyzed tissue type, i.e., “Tumor” and “Surrounding” tissue. The mean normalized expression levels of these genes, along with their standard errors of the mean (SEM) for each tissue type (tumor and adjacent tissue), are presented in Fig. 5. To evaluate differences in gene expression between tumor and adjacent tissues, we conducted a gene-wise Mann–Whitney U test [23]. Statistical significance was annotated as follows. Genes with *p*-values between 0.01 and 0.05 are marked with ‘*’, with *p-*values between 0.001 and 0.01 are denoted by ‘**’, and with *p*-values below 0.001 are indicated by ‘***’. No statistically significant are labeled as ‘n.s’.

## Results

### Chronic CRD results in abnormal mammary gland morphology

**Fig. 1:**
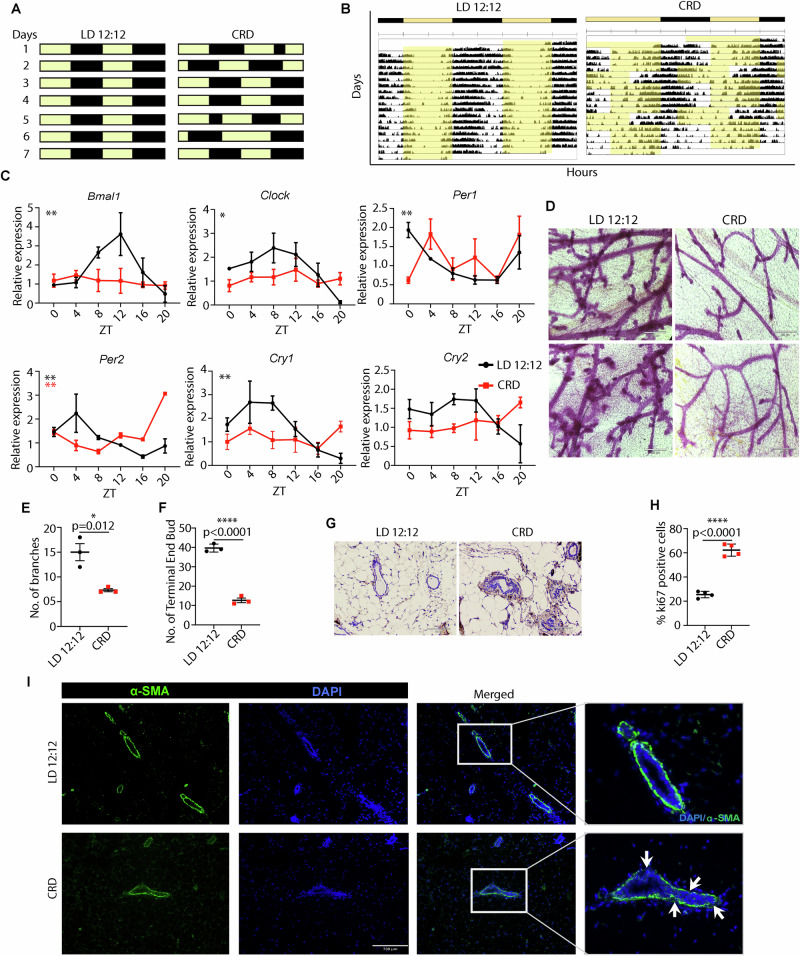
CRD severely impairs rhythmicity and disrupts mammary gland morphogenesis. **A** Schematic representation of the CRD protocol. **B** Representative actograms of LD and CRD mice. The FVB mice were randomly assigned to either the LD or CRD protocol for four weeks before being subjected to running-wheel recording for three weeks. LD: 12 h light and 12 h dark; CRD: circadian rhythm disruption using jet lag protocol, represented by a shortening of the dark period by 8 h every third day. **C** The female mice were housed in either normal light and dark (LD12:12) or CRD conditions for 8 weeks before tissues were collected every 4 h over 24 h. The timing of light-dark transitions on the day of collection was the same for at least 24 h before the collection. Mammary glands were collected at the indicated times. Gene expression normalized to *Gapdh* expression was measured using quantitative real-time PCR data represented as mean ± SEM for *n* = 3 females per time point and light condition. Rhythmicity was determined using JTK_Cycle analyses; *pJTKcycle < 0.05, **pJTKcycle < 0.01, ***pJTKcycle < 0.001, and ****pJTKcycle < 0.0001. CRD disrupts branching morphogenesis in WT virgin FVB mice. **D–F** Representative images of branching and development of terminal end buds in LD and CRD-induced mammary glands. scale bar: 350 μm. The effect of CRD on mammary ductal hyperplasia is shown by hematoxylin and eosin (H&E) staining of the mammary gland, (Scale bar: 100 μm) **G**, by using Ki67 staining **H**, and by immunofluorescence staining of α-SMA in the mammary gland (scale bar: 730 μm) **I**, white arrow indicates the disrupted myoepithelial layer. *p < 0.05, ****p < 0.0001 represents the significance level from an unpaired *t*-test.

The jet lag paradigm mimics the CRD that humans face during shift work [24] or travel across time zones. Mice were exposed to CRD conditions every 2 days (Fig. 1A). Friend leukemia virus B (FVB) mice were housed under normal light and dark (LD 12:12), or CRD conditions for 8 weeks before the mammary glands were collected every 4 h for 24 h. The disruption of rhythm was assessed by the locomotor activity (wheel-running assay) (Fig. 1B). CRD did not significantly impact the weight of mice (Fig. S1A). Real-time PCR analysis showed that CRD led to loss of rhythmicity (*p*JTKcycle < 0.05) for most genes, including *Cry1*, *Cry2*, *Bmal1*, *Clock*, and *Per1* (Fig. 1C) but not for *Per2* (Fig. 1C), suggesting the desynchronization of the core clock components. To understand the effect of CRD on the mammary gland, we examined whole-mount preparations of inguinal mammary glands from LD and CRD-induced mice. Chronic CRD decreased branching (bifurcations) (Fig. 1D, upper panel, 1E, S1B) and the number of terminal end buds (TEBs) (Fig. 1D, lower panel, 1 F, S1B) in the mammary glands. Compared to the mammary glands of LD mice (control), CRD-induced mice exhibited a higher frequency of ductal hyperplasia, as evidenced by hematoxylin & eosin (H&E) staining (Fig. 1G) and immunohistochemical analysis of Ki67 expression (Figs. 1H, S1C). The expression of α-smooth muscle actin (α-SMA) showed that the myoepithelial cell layer at the ductal region is disrupted in the CRD-induced mammary glands compared to LD-induced mammary glands (Fig. 1I), which signifies a disruption in the protective cell layer surrounding the milk ducts, which can be an indicator of early signs of breast cancer [25, 26].

### Chronic CRD accelerates aggressive mammary tumorigenesis and lung metastasis

**Fig. 2:**
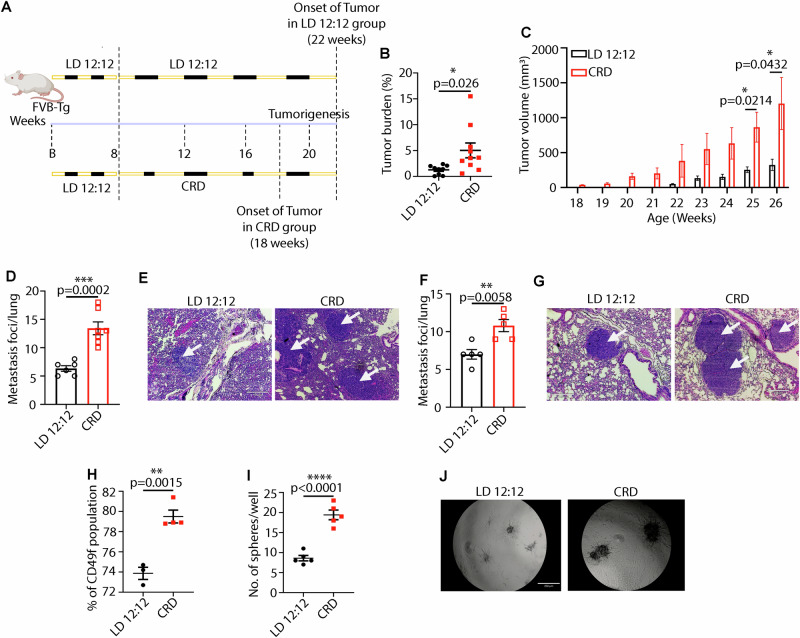
CRD increases aggressive mammary tumorigenesis and lung metastasis. Experimental timeline for evaluation of the effect of CRD on spontaneous TNBC in (FVB-Tg(C3-1-TAg)cJeg (C3-TAg) mice **A**. **B** Tumor burden (tumor to body weight ratio) as % in LD (*n* = 9) or CRD (*n* = 10) conditions. Column data represent the mean ± SEM values for individual animals. The effect of CRD on tumor initiation is shown in **C**. Number of metastatic foci in the lungs of LD (*n* = 6) and CRD (*n* = 7) mice at the time of their sacrifice is shown in **D**. *p-*value obtained from a binomial two-sided test. Indicated (n) represents the number of independent experiments as biological replicates. Metastatic foci in the lungs (white arrows) are shown using H&E staining, scale bar 150 μm **E**. CRD-induced lung metastasis in 4T1 tumor model in BALB/cJ mice *n* = 5 as shown by the graphical representation **F** and H&E staining (white arrows) **G**, scale bar 100 μm. **H** The percentage of CD49f-breast cancer stem cell (CSC) subpopulations in LD and CRD tumors is shown as a graph. Results represent the mean ± SEM of 3–4 independent experiments. *p*-value obtained from an unpaired two-sided *t*-test. Mammosphere formation efficiency (MFE%) of LD and CRD tumor cells (*n* = 5) is shown in Figure **I** with lines indicating the mean ± SEM. *p*-value obtained from an unpaired two-sided *t*-test. The formation of organoids (tumoroids) from LD and CRD tumors is shown in the **J** Scale bar, 250 μm. *p < 0.05, **p < 0.01, ***p < 0.001, ****p < 0.0001 represents the significance level from an unpaired *t*-test.

To investigate the effect of CRD on aggressive basal tumorigenesis, we used a genetically engineered mouse model (GEMM) of human basal tumors [27]. Female hemizygous mice develop mammary gland adenocarcinomas at approximately 24 weeks of age [27]. The mice were placed under LD or CRD conditions when they were 8 weeks old and maintained through the end of the study at 27 weeks (Fig. 2A). Because of the highly aggressive tumors in CRD-induced mice, we euthanized the animals at 27 weeks. CRD did not significantly impact the weight of mice (Fig. S2A). Peripheral blood cell counts (Fig. S2B, S2C) and blood chemistry (Fig. S2D–F) showed no significant differences between those two groups. However, the albumin-to-globulin ratio (AGR) was decreased significantly (Fig. S2G). A high AGR shows a good prognosis for survival in different solid-tumor patients [28]. Therefore, CRD negatively affects cancer prognosis by decreasing the AGR. Single-cell RNAseq (scRNA-seq) (Fig. S3A) and real-time PCR (Fig. S3B) of tumor samples showed that at the time of harvesting (ZT 12 for LD mice, for CRD-induced mice, ZT was not followed), CRD did not substantially alter the expression of the clock genes, *Per1*, *Per2*, *Clock*, and *Cry2*, whereas *Cry1* expression increased significantly in CRD-induced tumors (Fig. S3B).

CRD significantly increased mammary tumor burden (Fig. 2B) and accelerated tumor initiation, with tumors appearing by 18 weeks (Fig. 2C) compared to the 22 weeks in LD animals. However, no difference was observed in the spectrum of tumor grades assessed using histopathology [29] between the two groups, with most tumors being grade 2 (Fig. S4A, B). The number of metastatic foci in the lungs was significantly higher in the CRD-induced mice (Fig. 2D, E). To investigate the impact of CRD on tumor progression, 4T1 cells were introduced into the female BALB/cJ mice via orthotopic transplantation into the 4th mammary gland. When mice developed palpable tumors, they were placed into LD or CRD conditions for 3 weeks. These data suggest that CRD has a limited impact on mammary tumor progression (Fig. S4C). However, we did observe a significant increase in the formation of lung metastasis foci (Fig. 2F, G). We observed a significant increase in cancer stem cells (CSCs) expressing CD49f marker (Figs. 2H, S4D) in CRD tumors via flow cytometry, which was further confirmed by mammosphere assay. CRD-induced tumor cells had a significantly higher mammosphere formation efficiency than LD tumor cells (Figs. 2I, S4E). Recent studies have used three-dimensional (3D) organoids as in vitro organs, with numerous applications ranging from disease modeling to drug screening [30]. Our organoid study showed that CRD tumors exhibited more aggressive organoids than LD tumors (Fig. 2J).

### Circadian desynchronization alters the TME

**Fig. 3:**
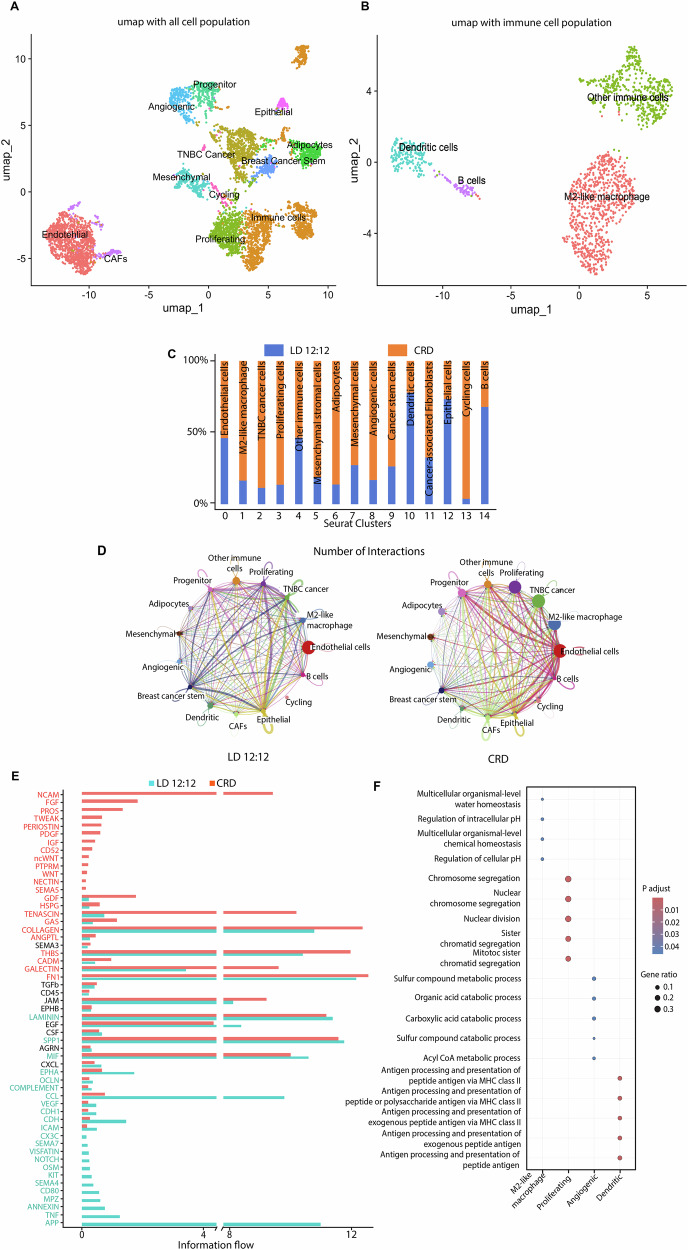
CRD alters the tumor microenvironment. Cell clusters from 10x Genomics scRNA-seq analysis visualized using Uniform Manifold Approximation (UMAP). UMAP plot shows different cell population **A**. UMAP visualization of reclustered immune cells revealing different populations of immune cells **B**. Fraction of cells from tumors (LD and CRD) in each cluster **C**. Clusters were annotated for their cell types as predicted using canonical markers and signature-based annotation with Garnett. Expression markers for endothelial cells (*Cd34*, *Cd31*), M2-like macrophages (*Fizz1, CD206, Tgm2, CD45*), TNBC cancer cells (*Trps1, Mmp2, Hspb1*), proliferating cells (*Cdk1, Pcna, Ki67)*, other immune cells (*Fth1, Cd45, Cd33, Cd5, Cd11c, Cd68, Tgm2, Fizz1)*, progenitor cells (*Nes, Cd48*), adipocytes (*Tmem26, Fabp4, Hoxc9*), mesenchymal cells (*S100a4, S100a6, Cd146*), angiogenic cells (*Rpl35a, Ang2, Tie1*), breast cancer stem cells *(Fxyd3, Aldh1, Lgr4*), dendritic cells (*Cd11c, Cd103, Trem1*), cancer-associated fibroblast (*Col1A1, Col1A2, Cd90*), epithelial cells (*Epcam, Cdh1, Ket14*), cycling cells (*Cdk4, Mcm2, Cdk6, Cd68, Cd90*), and B cells (*Cd83, Cd86, Cd19*). The comparison of the total number of interactions among different cell populations between LD and CRD tumors is shown in **D**. Edge width is proportional to the number of interactions, which assesses the number of ligand–receptor pairs contributing to the communication between two interacting cell populations. CellChat analysis quantitatively analyzes intercellular communication networks **E**, Green: LD12:12, Red: CRD. The analysis shows differential ligand–receptor interactions between LD and CRD tumors. Significant signaling pathways were ranked based on differences in the information flow within the inferred networks between LD and CRD tumors. Dot plot profiling of the Gene Ontology (GO) analyses in each cluster **F**. The dot plot represents the average expression of GOs per cluster. The color gradient of dots represents the expression level, whereas the size represents the percentage of cells expressing any genes per cluster. The number indicates the total number of genes identified belonging to GO pathways significantly enriched in the cluster.

To investigate the TME, scRNA analysis was performed on cells isolated from LD and CRD-induced tumors. Single cells were isolated from the tumors without surface marker selection. Using proper quality control (QC) and filtration, we obtained single-cell transcriptomic data for the studied tumors (*N* = 2/set). A total of 5804 QC-positive single cells (LD and CRD tumors) were clustered according to their expression profiles (Fig. 3A), and 20,764 genes were identified. Different cell populations (Fig. 3A) were clustered using the Seurat package based on specific cell markers as shown by the dot plot (Fig. S5F). To better characterize the immune cell subtypes, we re-clustered the total immune cell population at higher resolution, and identified four distinct subclusters (Fig. 3B). Using the established expression markers [31–73], we found that CRD altered different cell populations in the TME (Fig. 3C). CRD induced a higher number of M2-like macrophage, TNBC cancer cells, cycling cells, adipocytes, cancer-associated fibroblasts (CAFs), and proliferating cells but a lower number of B cells, and dendritic cells (DCs) in the tumor (Fig. 3C). Cell-cell communication was investigated using CellChat, which showed the differences in the total number of ligand-receptor (L-R) interactions between different cell types in the tumors (Fig. 3D). For example, the interaction between CAF with proliferating cells, TNBC cells, and M2-like macrophages increased under CRD conditions (Fig. 3D). We identified 3429 and 3992 critical ligand-receptor pairings (Fig. S5A) and their strength (Fig. S5B) in LD and CRD tumors, respectively. CellChat also identified signaling pathways linked to ligand–receptor interactions (Fig. 3E). Specific signaling pathways, including those involving fibroblast growth factor (FGF), noncanonical WNT, and platelet-derived growth factor (PDGF), were exclusively detected in CRD tumors, whereas Oncostatin M, and NOTCH, were detected solely in LD tumors. The signaling pathways, such as transforming growth factor β (TGFβ) and epidermal growth factor (EGF), were detected in both LD and CRD tumors (Fig. 3E). Gene Ontology (GO) analysis (Fig. 3F) indicated that genes associated with the acyl CoA metabolic process, nuclear division, regulation of cellular pH, carboxylic acid catabolic process were upregulated in CRD.

### CRD induces immunosuppressive TME and turns tumors “cold”

Tumors are “hot” when they show signs of inflammation, characterized by an infiltration of T cells mobilizing to fight the cancerous cells, whereas non-immunogenic “cold” tumors lack infiltrating T cells, which makes it challenging to provoke an immune response with immunotherapy drugs [74]. To characterize different immune cell populations in the TME of LD and CRD-induced tumors, scRNA-seq, flow cytometry, and multiplex immunostaining (MxIF) were performed. scRNA-seq with CRD-induced tumors showed that CRD reduced CD3^+^T cells infiltration (Fig. S5C) and enhanced regulatory T-cell (T_reg_) infiltration (Fig. S5D). CRD enhanced M2-like macrophage (Fizz1, CD206, Arg1) [35, 75] populations while decreasing M1-like macrophage (CD80, CD38, Nos2) [35, 75] populations (Fig. S5E).

**Fig. 4:**
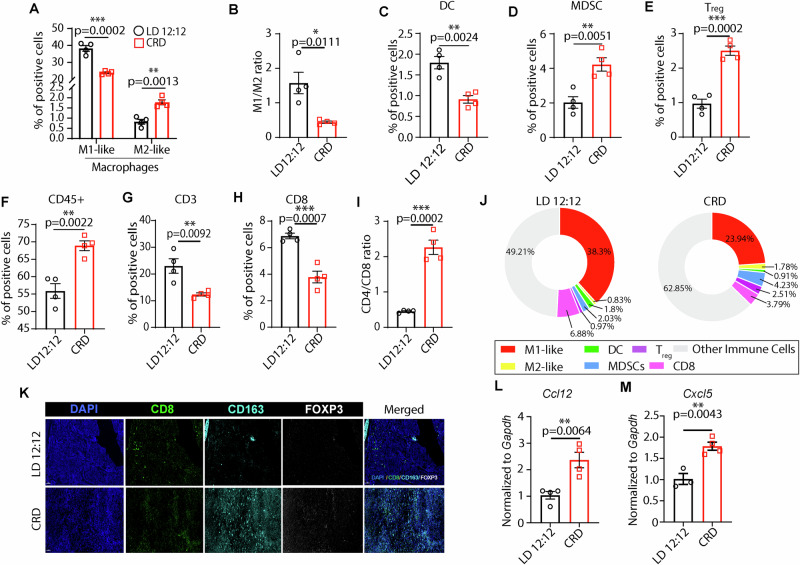
CRD turns tumors “cold”. Flow cytometry study showed the TME of LD and CRD-induced tumors. The percentage of antitumor M1-like macrophage (CD45^+^CD11b^+^CD86^+^) population was decreased, and pro-tumor M2-like macrophage (CD45^+^CD11b^+^CD163^+^CD206^+^) population was increased in CRD-induced tumors compared to LD tumors **A**, resulting in an overall decrease in M1/M2 ratio **B**. CRD reduced DC population significantly **C**, whereas enhanced MDSC population in the tumors **D**. A significant increase in the FoxP3^+^T_reg_ population was observed in the CRD-induced tumors **E**. The percentage of total leukocytes (CD45^+^ cells) in LD and CRD-induced tumors is shown in **F**. CRD decreased CD3^+^T population significantly in tumors **G**. The percentage of cytotoxic T cells (CD8^+^T cells) is decreased in CRD-induced tumors as shown in **H** and the CD4/CD8 ratio was increased in CRD-induced tumors **I**. Donut chart representing the percentage of immune cells in the TME of LD and CRD-induced tumors **J**. All the representative graph for flow cytometry is *n* = 4. *p*-value obtained from an unpaired two-sided *t*-test. Representative multiplexing immunostaining image (MxIF) of cytotoxic CD8 T cell (CD8, green), M2-like macrophage (CD163, cyan) T_reg_ (FOXP3, white) in the LD and CRD-induced tumors (*n* = 4), Scale bar= 50 μm **K**. The expression of chemokines *Ccl12* **L** and *Cxcl5* **M**, is shown using real-time PCR in LD and CRD-induced tumors (*n* = 3-4). *p < 0.05, **p < 0.01, ***p < 0.001, ****p < 0.0001 represents the significance level from an unpaired *t*-test.

Flow cytometry analysis (FMO and gating strategy, Fig. S6A, B), with CRD-induced tumors supported the scRNA-seq data. CRD induced M2-like macrophage (pro-tumor) infiltration while reducing M1-like macrophage (anti-tumor) infiltration (Fig. 4A). Tumors from mice under CRD conditions exhibited a ratio (M1:M2) that favored M2-mediated immunosuppression (Fig. 4B) and facilitated tumor growth. CRD-induced tumors showed a lower DC population compared to LD-induced tumors (Fig. 4C). There was a significant increase in the MDSC population (Fig. 4D) in CRD-induced tumors. Our study showed a significant enrichment of the immunosuppressive T_reg_ (CD4^+^FoxP3^+^) population (Fig. 4E) in the CRD-induced tumors. CRD significantly enhanced the CD45^+^ immune cell population in tumors (Fig. 4F). CRD inhibited infiltrating CD3^+^T cells in the tumors (Fig. 4G). Our study showed a significant inhibition of the number of infiltrating CD8^+^T cells (Fig. 4H) and an increase in the CD4/CD8 ratio (Fig. 4I) in CRD-induced tumors. The increased CD4/CD8 ratio in CRD-induced tumors also indicates an immunosuppressive microenvironment. A donut chart shows the differences in different immune cell populations in LD vs. CRD-induced tumors (Fig. 4J). Our immunofluorescence assay showed that CD8^+^T-cell infiltration was decreased, but M2-like macrophages and T_reg_ expression were enhanced in CRD-induced tumors compared with LD-induced tumors (Fig. 4K), making the tumor “cold”. Altogether, our results confirmed the “cold” characteristics of CRD tumors.

Chemokines facilitate the immunosuppressive TME by enhancing the differentiation and infiltration of immunosuppressive cells, such as T_reg_ cells, MDSCs, etc. By using a Proteome Profiler, we noticed marked increases in circulating IFN-γ, IL-1β, G-CSF, and IL-16 levels in CRD mice (Fig. S7A). The chemokine/cytokine network, known to favor an immunosuppressive microenvironment [76], such as *Ccl12* (Fig. 4L) and *Cxcl5* (Fig. 4M), were upregulated in CRD-induced tumors. Levels of *Ccl28* (Fig. S7B, C), *Il-17β*, and *Il-10* (Fig. S7C) were upregulated modestly (but not significantly) in CRD-induced tumors.

### Malignant human breast cancer cells have high CRD

**Fig. 5:**
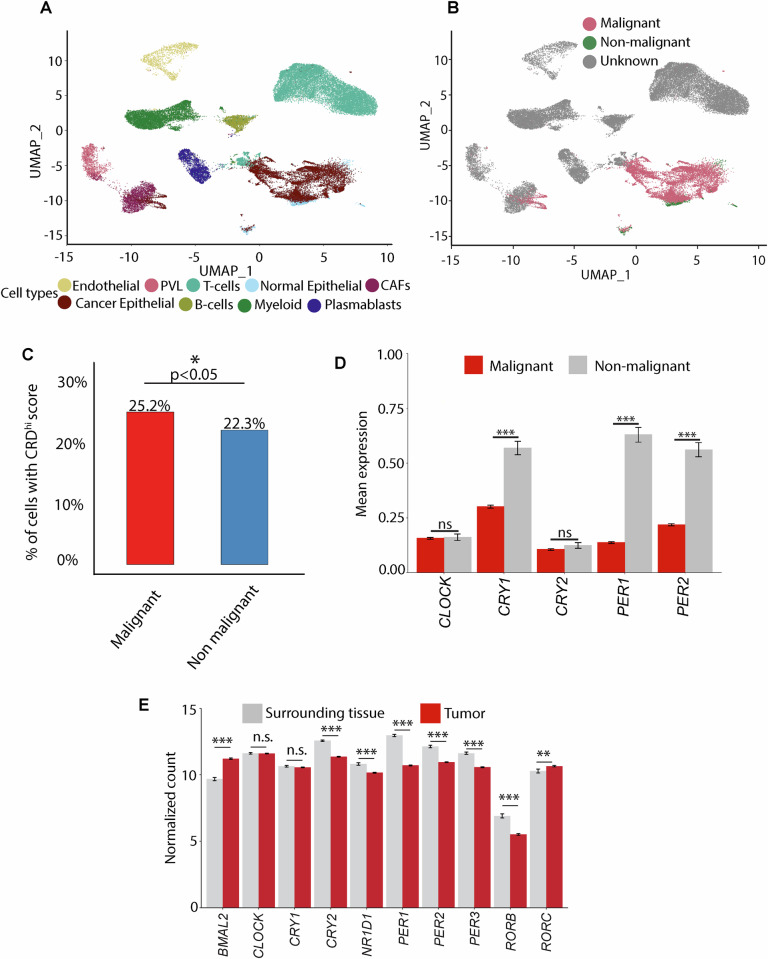
The human circadian clock is disrupted in breast cancer. UMAP embeddings corresponding to TNBC patients only after data harmonization colored by manually annotated cell types (total cells = 42,512) **A**, and by inferCNV cell types **B** [77]. Moving from the plot in **B**, 30,719 cells had an unknown inferCNV calls and 11,793 had a known inferCNV call. These 11,793 single cells across 9 TNBC patients were used for downstream analysis. The percentage of malignant and nonmalignant cells with CRD^high^ is shown in **C**. The mean expressions of the clock genes, along with the standard errors of the mean (SEM) for each cell type (malignant and non-malignant cells), are presented in **D. E** The mean expression of the clock genes (+/- standard error of mean) over patients by the type of tissue analyzed in TNBC tumors (*n* = 1104) and surrounding tissue (*n* = 113) using TCGA (Source file: UCSC Xena Database) [22]. Genes with *p*-values between 0.01 and 0.05 are marked with ‘*’, those with *p*-values between 0.001 and 0.01 are denoted by ‘**’, and genes with *p*-values below 0.001 are indicated by ‘***’. Genes exhibiting no statistically significant differential expression are labeled as ‘n.s’ (not significant).

Earlier studies [8] and our present study used GEMMs to investigate the effect of CRD on tumorigenesis. However, more insights are needed into the molecular mechanisms underlying CRD at the single-cell level and in human tumors. To investigate the process underlying CRD during mammary tumorigenesis, we used a scRNA sequence dataset from a previously published study [77]. The dataset included TNBC, HER2-positive, and luminal tumors. We ran UMAP corresponding to the TNBC patients colored by manually annotated cell types (Fig. 5A). Using large-scale copy number variation (CNV) from the transcriptomics data [78], we separated definitive malignant cells from potential nonmalignant cell populations in TNBC (Fig. 5B), luminal (ER^+^/PR^+^) (Fig. S8A), and HER2^+^ tumors (Fig. S8B). Based on a previous study [79], the CRD score was calculated using the expression of 358 circadian-related genes [79]. Next, we quantified the CRD scores of malignant and nonmalignant cells in different types of human breast tumors. Aggressive TNBC comprised significantly (*p* < 0.05) more malignant cells with high CRD scores (Fig. 5C) than luminal (Fig. S8C) and HER2^+^ tumors (Fig. S8D). *CRY1*, *PER1*, and *PER2* expression decreased in TNBC malignant cells compared to non-malignant cells, as shown in Fig. 5D To define the role of CRD in patient populations, we used RNA-seq data from tumors and surrounding tissues of breast cancer (BRCA) patients in the Cancer Genome Atlas (TCGA) [22] (https://github.com/Arhit-Chakrabarti/TCGA-BRCA-Analysis). The expression of core clock genes such as *CRY2*, *RORB*, *NR1D1*, *PER1, PER2, and PER3* was significantly lower in the tumors than in the surrounding tissues (Fig. 5E). The expression of *BMAL2*, and *RORC* was increased significantly, whereas *CLOCK* and *CRY1* expression remain unchanged (Fig. 5E). These data suggest that the core clock is disrupted in human breast tumors.

### CRD creates “cold” tumors by inducing LILRB4a expression

**Fig. 6:**
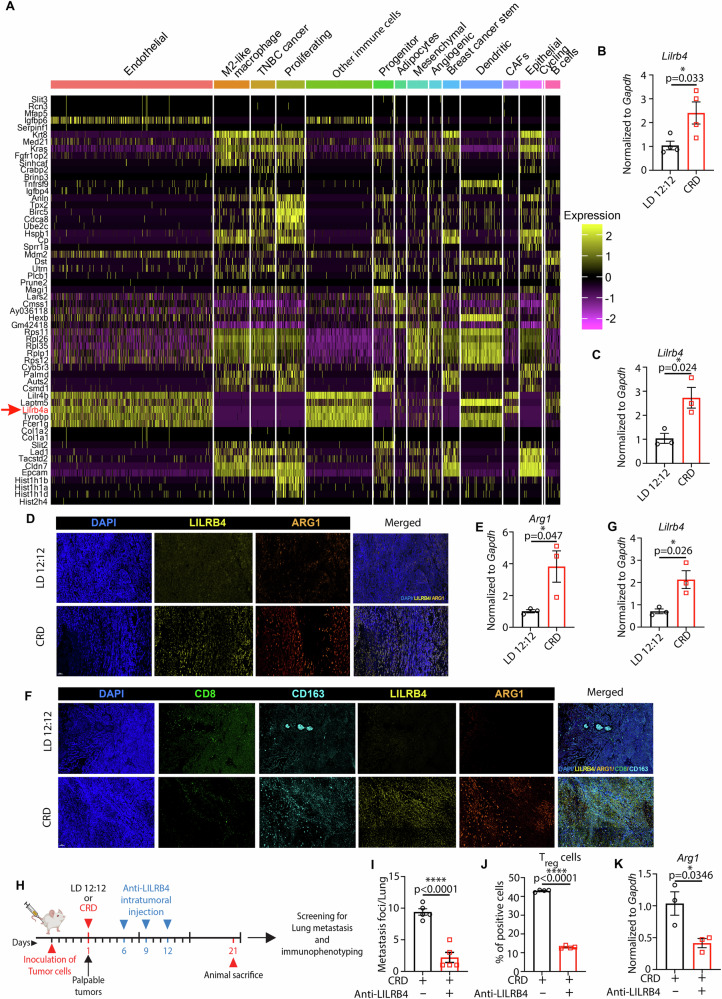
CRD enhances LILRB4a expression to create an immunosuppressive tumor microenvironment. **A** Heatmap of differentially expressed genes in CRD-induced tumors identified using DESeq2 analyses of scRNA-seq. The transcript level of *Lilrb4* was analyzed in LD and CRD-induced GEMM tumors (*n* = 4) **B** and 4T1 tumors (*n* = 3) **C** using real-time PCR. **D** The expression of LILRB4 (yellow), arginase 1 (ARG1, a downstream target of LILRB4a) (orange) was analyzed using immunofluorescence. Scale bar: 50 μm **E** The transcript level of *Arg1* in LD and CRD-induced GEMM tumors was analyzed using real-time PCR. (*n* = 3) **F** Representative MxIF showing the expression of LILRB4, CD8, CD163, and FOXP3 markers in LD and CRD-induced GEMM tumors. Scale bar: 50 μm. **G** The transcript level of *Lilrb4* in LD and CRD-induced mammary glands was analyzed using real-time PCR. (*n* = 3) **H** 4T1 cells were injected into the mammary fat pads of BALB/cJ mice for the LILRB4-targeted immunotherapy. Briefly, 4T1 cells were orthotopically transplanted into BALB/cJ mice, once palpable tumors developed (4-5 days), mice were placed into LD or CRD condition. The LILRB4 antibody was administered on days 6, 9, and 12 after developing palpable tumors. Mice were sacrificed after 3 weeks (day 21) (Fig. 5H), and lung metastasis and the TME were examined. The number of metastatic foci in the lungs of CRD (control) and CRD (LILRB4-antibody) mice (*n* = 5) at the time of their sacrifice is shown in **I**. The percentage of T_reg_ cells in control and LILRB4-antibody-treated CRD-induced tumors (*n* = 4) is shown in **J**. The transcript level of *Arg1* in control and LILRB4-antibody-treated CRD-induced tumors is shown by real-time PCR (*n* = 3) **(K)**. *p < 0.05, ****p < 0.0001 represents the significance level from an unpaired *t*-test.

Differential gene expression (DGE) analysis of our scRNA-seq data revealed that *Lilrb4a* (*Lilrb4*) was upregulated in the CRD-induced tumor samples (Figs. 6A, S9A). LILRB4, an immunoreceptor tyrosine-based inhibitory motif-containing receptor, suppresses T-cell activity via a signaling pathway involving apolipoprotein E (APOE), SH2-domain-containing protein tyrosine phosphatase-2 (SHP-2), urokinase-like plasminogen activator (uPAR), and arginase 1 (ARG-1) (*29*). Recent studies have shown that LILRB4 creates an immunosuppressive microenvironment in AML [80] and solid cancers [18] by inhibiting CD8^+^ T-cell infiltration and inducing T_reg_. The DGE analysis showed an upregulation of *Lilrb4* in endothelial cells along with CAFs, B cells, other immune cells, and some DCs (Fig. 6A). Real-time PCR showed that CRD enhanced *Lilrb4* levels significantly in CRD-induced tumors (Fig. 6B, C). ARG1 expression was increased significantly in CRD-induced tumors, as shown by immunofluorescence (IF) assay (Fig. 6D) and real-time PCR (Fig. 6E). LILRB4-associated factors such as *Arg1, uPar (Plaur), Cxcl5*, and *Ccl12* follow a similar pattern as their expression is high in endothelial cells, dendritic cells, and other immune cells (Fig. S9B). The CD8 infiltration decreased, whereas M2-like macrophage populations (CD163) increased along with LILRB4 in CRD-induced mammary tumors, as shown using MxIF (Fig. 6F). Furthermore, *Lilrb4* transcript levels were significantly increased in CRD-induced mammary glands (Fig. 6G), but did not exhibit any diurnal oscillations under LD and CRD conditions (Fig. S9C). The transcript level of *Arg1* increased modestly but non-significantly in the CRD-induced mammary glands (Fig. S9D). The transcript level of *Ccl12* increased significantly in CRD-induced mammary glands (Fig. S9E). The transcript level of *Cxcl5* is raised modestly but not significantly in CRD-induced mammary glands (Fig. S9F). Overall, these changes in the immune milieus of the CRD-induced mammary microenvironment can decrease adaptive immunity and enhance susceptibility to cancer and other diseases.

To investigate whether LILRB4 expression was responsible for creating an immunosuppressive TME, we explored the effects of LILRB4-immunotherapy on tumor progression in CRD mice by treating them with an anti-LILRB4 antibody [18]. Tumor volumes were observed to be invariable across the treatment groups (Fig. S9G), but we observed a significant decrease (*p* < 0.0001) in the prevalence of lung metastasis in anti-LILRB4-antibody-treated mice under CRD (Fig. 6H, I). Flow cytometric analysis showed an inhibition of the T_reg_ cell population in LILRB4-antibody-treated tumors under CRD conditions (Fig. 6J). LILRB4-targeted therapy inhibited *Arg1* transcript levels (Fig. 6K) in CRD-induced tumors. We also observed a modest but non-significant decrease in *Ccl12* (Fig. S9H) and *Cxcl5* (Fig. S9I) transcript levels in anti-LILRB4-antibody-treated CRD-induced tumors. No significant difference was observed in the number of lung metastasis foci in LD mice treated with the anti-LILRB4antibody (Fig. S9J). Anti-LILRB4-antibody treatment under LD conditions did not decrease the T_reg_ population (Fig. S9K), *Arg1* transcript level (Fig. S9L), and the chemokines (*Ccl12, Cxcl5*) (Fig. S9M, N) significantly. These findings fortify the notion that elevated LILRB4 contributes to CRD-induced aggressive tumorigenesis.

### LILRB4 regulates CRD-induced tumorigenesis via a non-canonical WNT signaling pathway

**Fig. 7:**
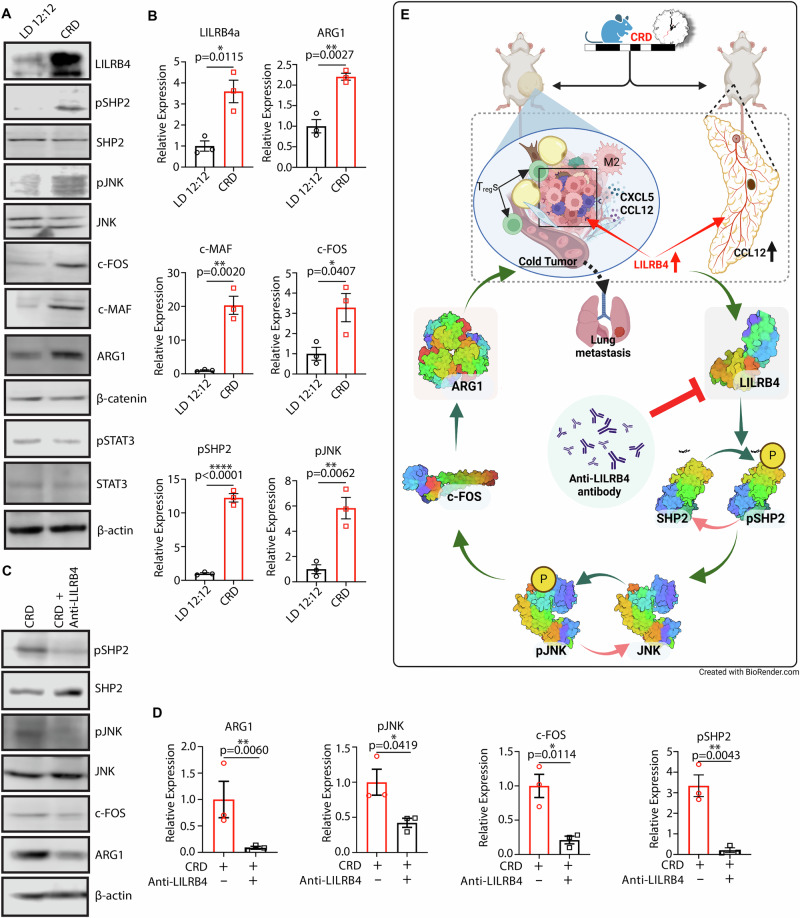
LILRB4 regulates CRD-induced mammary tumorigenesis via non-canonical WNT signaling pathway. **A** Western blot analysis showing the expression of LILRB4 and downstream signaling molecules, including p-SHP2, SHP2, p-JNK, JNK, β-catenin, p-STAT3, STAT3, c-FOS, c-MAF, and ARG1, in LD 12:12 and CRD-induced tumors. **B** The densitometric analyses comparing the protein expressions relative to β-actin. The results show data as mean from three independent experiments (*n* = 3). **C** Western blot validation of the inhibitory effect of anti-LILRB4 antibody treatment on non-canonical WNT signaling pathway in CRD-treated tumors. β-actin serves as a loading control. **D** The expressions of ARG1, pSHP2, pJNK, and c-FOS following the anti-LILRB4 antibody was represented as relative expression to β-actin. (*n* = 3) **E** Diagrammatic representation of the molecular mechanism of CRD-induced immunosuppressive microenvironment in breast cancer. CRD enhances aggressive TNBC and lung metastasis by creating an immunosuppressive tumor microenvironment. CRD also disrupts the mammary gland morphology. Our study revealed that CRD enhanced LILRB4 expression, which induces an immunosuppressive “cold” TME by increasing the M2-like macrophage and T_reg_ populations and decreasing M1-like macrophage infiltration. Inhibition of LILRB4 via LILRB4-targeted antibody alleviates CRD-induced immunosuppressive TME and inhibits lung metastasis, by reducing phosphorylation of SHP2 and JNK and suppressing downstream pro-metastatic effectors such as c-FOS and ARG1. *p < 0.05, **p < 0.01, ***p < 0.001, ****p < 0.0001 represents the significance level from an unpaired *t*-test.

Using the CellChat tool that quantitatively inferred and analyzed intercellular communication networks from scRNA-seq data, our study identified some signaling pathways in the LD and CRD-induced tumor samples. This analysis showed an upregulation of the noncanonical WNT pathway in CRD-induced tumors (Fig. 3E). Earlier studies showed that LILRB4 regulates melanoma [81], multiple myeloma [82], and leukemia [80], via activation of STAT3 and inhibition of NF-κB [81]. We found that although SHP2 phosphorylation is elevated in CRD-induced tumors, however, the STAT3 signaling pathway is not altered under CRD. We found that JNK is activated (pJNK) in CRD-induced tumors, which in turn activates the downstream target c-FOS in CRD-induced tumors (Figs. 7A, B, S10A). c-FOS was found to induce c-MAF [83, 84], which enhanced ARG1 in CRD-induced tumors. LILRB4-induced ARG1 activation was inhibited in the mice treated with LILRB4-targeted immunotherapy by inhibiting the noncanonical WNT pathway in CRD-induced tumors (Figs. 7C, D, S10B). Together, we identified that LILRB4 regulates CRD-induced tumorigenesis and lung metastasis via the noncanonical WNT signaling pathway (Fig. 7E).

## Discussion

Following the chronic jet lag protocol, we showed that CRD interrupted the biological clock, affected the mammary gland morphology, accelerated aggressive basal mammary tumorigenesis and lung metastasis by altering the TME. CRD induced an immunosuppressive TME and turned the tumors “cold.” We also identified the molecular mechanism underlying CRD-induced aggressive mammary tumorigenesis.

We found that CRD-induced mammary glands exhibited reduced TEBs and branching morphology. Earlier studies showed that circadian clock genes may play a role in mouse mammary gland development and differentiation [85], and disruption of clock genes can cause abnormal mammary gland development [86, 87]. Although environmental circadian disruption caused by shiftwork, or jet lag can significantly affect the expression of numerous circadian genes, disrupting the normal rhythmic pattern of gene activity within the body. However, its role in mammary gland and aggressive mammary tumorigenesis has not yet been studied. CRD-induced mice showed mammary gland ductal hyperplasia, which can be an early sign of ductal cancer [88]. This indicates the potential role of CRD in breast cancer initiation.

Our study suggests that CRD may influence the early events of tumor initiation and lung metastasis but appears to have a minimal impact on the progression of established tumors. LILRB4 expression was increased in mammary glands and CRD-induced mammary tumors, revealing an unidentified mechanism responsible for CRD-induced tumorigenesis, lung metastasis, and mammary gland abnormality. The jet lag protocol used here mimics the effects of rotating shift work or frequent eastbound trans-meridian flights and has been previously shown to cause severe perturbations of circadian rhythmicity [6, 8, 19]. We found that CRD affected the rhythmicity of many clock genes in the mammary glands; however, *Per2* expression remained rhythmic after CRD, indicating that *Per2* is more sensitive to phase entrainment signals of molecular oscillators in peripheral tissues.

Using scRNA-seq and TCGA data, we showed that the expression of certain circadian genes was decreased in aggressive human TNBCs compared with that in the surrounding mammary tissues. Our analysis also revealed that the malignant cells in aggressive human TNBCs had higher CRD scores and downregulated *PER1*, *PER2*, and *CRY1* significantly compared to nonmalignant cells.

Our study showed that CRD induced an immunosuppressive tumor microenvironment, which could be involved in increased tumor burden in mice maintained under the CRD schedule. The TME consists of natural killer cells, CD8^+^ T cells, proinflammatory macrophages (M1), and DCs, which elicit antitumor immune responses, whereas the presence of MDSCs and FOXP3^+^ T_reg_ counteracts tumor immunity [8]. Our scRNA-seq study and flow cytometry showed that CRD decreased CD3^+^ T-cell infiltration and the DC population. This supports the recent finding that DC and T-cell-autonomous circadian clocks are responsible for time-of-the-day-dependent antitumor effects [89]. We found an increase in the M2-like macrophage population and a decrease in the M1-like macrophage population, which creates an immunosuppressive microenvironment [90] in CRD tumors. CRD was found to inhibit CD3^+^ T cell population, which is known to activate cytotoxic CD8^+^ T cells [91]. CD3^+^ T cells are known to have anti-tumor properties and are associated with better patient survival [92]. CRD increased the leukocyte (CD45^+^) population, corroborating that the jet lag schedule enhances leukocyte levels in melanoma [93].

Our scRNA-seq and real-time PCR studies showed that CRD induced a pro-tumorigenic and immunosuppressive microenvironment by increasing the expression of *Ccl12* and *Cxcl5*. The upregulation of *Ccl12* (human orthologs of CCL2) [94] and *Cxcl5* reportedly leads to the accumulation of MDSCs [95], which creates an immunosuppressive environment by suppressing CD8^+^T-cell infiltration [95]. CRD also enhances *Ccl28* production, which leads to the recruitment of T_reg_ cells in the TME [96].

We further demonstrated that CRD enhanced the expression of LILRB4 in vivo in healthy mammary glands and mammary tumors. LILRB4 is expressed on endothelial cells along with various immune cells, including microglia, monocytes, macrophages, DCs, T cells, neutrophils, and plasma cells, where it mainly acts as immunosuppressive receptor via ITIMs involved in the inhibition of cytokine production and suppression of T cell activity [97]. Recent studies have shown that LILRB4 creates an immunosuppressive microenvironment in AML [80] and solid cancers [18] by inhibiting CD8^+^ T-cell infiltration and inducing T_reg_. In different malignant cancers, LILRB4 expression in MDSCs is associated with decreased survival in patients [98]. Another study showed that LILRB4 blockade increased the proportions of tumor immune infiltrates, effector T (T_eff_) levels, and altered the TME toward reduced immunosuppression in solid tumors [18]. Together, these findings reveal that LILRB4 creates an immunosuppressive microenvironment, thereby decreasing its antitumor efficacy. Our study showed that CRD activated LILRB4 signaling in healthy mammary glands and aggressive TNBCs. Our scRNA-seq analysis revealed high expression of *Lilrb4* in endothelial cells, as well as in B cells, CAFs, a subset of dendritic cells and other immune cells. Notably, elevated LILRB4 levels on endothelial cells have been shown to promote immune tolerance and suppress nearby immune cell activity [99, 100]. Similarly, LILRB4 expression on DCs drives the differentiation of naïve T cells into regulatory T cells (T_regs_) and T suppressor cells (Ts), contributing to an immunosuppressive tumor microenvironment (TME) [100, 101]. Overall, this heightened LILRB4 expression enhances the T_reg_ population while suppressing cytotoxic T cells, thereby fostering an immunosuppressive microenvironment. Using real-time PCR and immunofluorescence analysis, we observed increased ARG1 expression in CRD-induced tumors. Analysis of the TME using MxIF showed an immunosuppressive microenvironment as the cytotoxic T cell and M1-like macrophage populations decreased. In contrast, T_reg_ and M2-like macrophage populations were increased in CRD tumors. We found that the disruption of circadian rhythm upregulated LILRB4 expression, which correlated with mammary tumor progression and abnormal morphology in the mammary glands.

CRD upregulated the expression of immunosuppressive chemokines (*Ccl12*) in mouse mammary glands, which is known to elevate the cancer risk [102]. An immunosuppressive environment can increase susceptibility to opportunistic infections [103] in CRD-induced patients. This study demonstrated how an inhibitory immune receptor altered the TME and influenced aggressive mammary tumorigenesis in response to CRD. We showed that the targeted LILRB4 immunotherapy reduced CRD-induced lung metastasis by inhibiting immunosuppressive TME. In addition to that, our study showed that unlike AML [80], melanoma [18, 81], and multiple myeloma [82], where LILRB4 has been shown to support tumor progression by activating STAT3 and inhibiting NF-κB, our study uncovered a new LILRB4 signaling mechanism that regulates CRD-induced mammary tumorigenesis via a noncanonical WNT signaling pathway via activation of JNK, c-FOS, c-MAF and the downstream target ARG1.

Our findings suggest that LILRB4 is a potential therapeutic target for mitigating CRD-induced cancer risk in populations exposed to chronic CRD, such as shift workers. LILRB4 inhibition was also found to improve the immunosuppressive microenvironment (Fig. 7E). However, how the disruption of the cell-autonomous molecular clock by CRD could enhance LILRB4 expression remains unknown. Additional investigations are required to determine whether the chronic elevation of LILRB4 levels in the mammary glands in response to CRD occurs early in the disease process and whether LILRB4 is present in other anatomical locations. Additional experiments are needed to investigate the reversibility of CRD-induced changes, determining the earliest time point at which morphological alterations can be detected, and assessing how long it takes for the mammary gland to return to a normal state following CRD exposure.

## Supplementary information

Ogunlusi o_supplementary

## Data Availability

All data needed to evaluate the conclusions in the paper are present in the paper and/or the Supplementary Materials.
