# Supplementary material for: LILRB4 regulates circadian disruption-induced mammary tumorigenesis via non-canonical WNT signaling pathway
Source: Oncogene. 2025 Oct 16;44(46):4491–504. doi: 10.1038/s41388-025-03597-5 (PMC12602328; doi:10.1038/s41388-025-03597-5)
Supplement: Supplementary file 1 — Ogunlusi o_supplementary [file 41388_2025_3597_MOESM1_ESM.pdf]

SUPPLEMENTAL MATERIALS and METHODS

A. Immunohistochemistry and immunofluorescence assay:

The formalin-fixed paraffin-embedded (FFPE) mouse mammary gland tissues were deparaffinized by incubating at 65°C for 2 hours. After antigen retrieval by boiling in sodium citrate buffer (pH 6.0) and blocking, the tissues were incubated overnight at 4°C with anti-ki67 polyclonal antibody (Abclonal, A16919) and α-Smooth Muscle Actin (ACTA2) polyclonal antibody (ABclonal, A1011). For immunohistochemistry, following incubation with the secondary antibody Biotinylated Goat Anti-Polyvalent (abcam, 70751) included in the IHC kit (IHC kit, abcam ab64264), the expression was visualized with DAB peroxidase substrate. The slides were counterstained with Mayer's Hematoxylin Solution and imaged under the Echo Revolve R4 microscope. The slides were stained with the secondary antibody, Alexa Fluor 488-conjugated anti-rabbit (Cell Signaling Technology, 4412S) for immunofluorescence. DAPI (ThermoFisher Scientific, D1306), in 1% BSA in PBS 0.1% Tween-20 was then used to counterstain the nucleus for 5 minutes. For the whole-mount staining, the mammary gland tissues were fixed using Carnoy's fixative solution, followed by overnight incubation with Carmine Alum Staining Solution. Stained tissues were mounted with Optic Mount I™ xylene mounting medium and observed under the microscope.

B. Flow cytometry:

The dissociated tumor cells (Table 1) were counted and resuspended in 1 ml of PBS . Zombie NIR™ Fixable Viability Kit (BioLegend, 423105) was applied to cells on ice for 20 min. After spinning down at 400 x g for 4 minutes, the cells were incubated with anti-mouse CD16/32 (Fc block) for 10 minutes on ice and fluorochrome-conjugated antibodies (Alexa Fluor® 700 anti-mouse CD4, Pacific Blue™ anti-mouse CD8a,

BV421 anti-mouse CD86, PE anti-mouse CD163, Alexa Fluor® 594 anti-mouse CD45, APC-Fire 810 anti-mouse Ly-6C , PE-Fire 810 anti-mouse Ly-6C, BV785 anti-mouse CD3, APC anti-mouse CD64, cFluor R685 anti-mouse CD11b, BV650 anti-mouse MHC Class II (I-A+I-E), SBV570 anti-mouse CD11c, BV605 anti-mouse CD206, and BV711 anti-mouse CD24) in the dark for 20 minutes. Cells were fixed and permeabilized using 2% paraformaldehyde and 0.1% Triton X-100 in PBS, respectively, and stained with anti-FoxP3 antibody (Alexa Fluor® 488 anti-mouse/rat/human FOXP3) for 20 minutes in the dark at 4°C. Afterward, stained cells were rinsed with FACS buffer (2% FBS in PBS) and kept in FACS buffer until analyzed using a Cytek Aurora spectral flow cytometer. Detailed antibody list provided as Table S3.

Table S1. The volume of tumors used in FACS study

LD tumors	Volume (mm^3)	CRD tumors	Volume (mm^3)
1	744.40275	1	711.16575
2	459.003625	2	539.990875
3	647.74525	3	544.348
4	452.8715	4	480.61225

C. Single-cell RNA sequencing (scRNA Seq):

We performed 2 sets of scRNA seq, with GEMM, where we compared the TME from tumor #1st set 507.84 mm^3 for LD, 578.36 mm^3 for CRD, and #2nd set: 519.067 mm^3 for LD, 749.048 mm^3 for CRD. The data in Fig. 3 used the first set of tumor samples (507.84 mm^3 for LD, 578.36 mm^3 for CRD). After brief washing in chilled sterile PBS, the tumors were kept in DMEM (ATCC, 30-2002), supplemented with 10% FBS (Sigma-Aldrich, F2442) and 1% antibiotic solution (Sigma Aldrich, P4333) on ice until minced and subsequently blended with gentleMACS Dissociator (Miltenyi Biotec, 130-093-235). MACS Miltenyi Tumor Dissociation Kit for mice (Miltenyi Biotech, 130-096-730) was used for further enzymatic digestion according to the manufacturer's

protocol. The suspension of cells was then transferred to the Texas A&M Institute for Genome Sciences and Society (TIGSS) for scRNA-seq. Single-cell sequencing libraries were prepared on the Chromium platform (10x Genomics) using the Single Cell 5' v2 Full Kit (PN-1000263). Cell type assignment was performed manually based on unique marker genes identified in each cell cluster from the UMAP reduction. Markers were cross-referenced with published literature specific to mouse mammary gland tissues. Differentially expressed genes were identified using the MAST [1] R package, and GO pathway enrichment analysis was performed with the clusterProfiler [2] R package. The CellChat R package analyzed cellular communication using default parameters. The most significant pathways were identified using the "rankNet" function and visualized with the "netVisual_diffInteraction" function.

D. **Human CRD analysis for breast cancer patients from scRNA seq data:**

All statistical analyses were performed using the R software package (version 4.3.0.). scRNA-seq data corresponding to the triple-negative breast cancer (TNBC) patients from Wu *et al.*, 2021[3] were pre-processed using standard steps outlined in Hao *et al.,* [4]. In particular, the features (genes) detected in at least 3 cells and cells showing expression of at least 200 features were considered for downstream analysis. The data were log-normalized by library size and multiplied by a scale factor 10,000. The data were further scaled such that the mean expression across cells is 0 and the variance across cells is 1. The UMAP plots were obtained after combining single cell data from all patients and performing data harmonization by different patients. The data harmonization was performed using Harmony (Korsunsky et al., 2019) [5]. The uniform manifold approximation and projection (UMAP) was performed on the processed data. The UMAP plot with cells clustered according to the inferCNV cell type. The cells which had no infer-CNV call are denoted as "unknown." To investigate

the level of circadian rhythm disruption in each cell, we calculated the CRD scores for the 358 circadian-related genes (CRGs) following He *et al.,* 2022 [6] separately for malignant and non-malignant cells. A cutoff of 75% based on quartiles was used as the threshold for the CRD level to segregate the scores as high (denoted as CRD^{hi}) and low (denoted as CRD^{low}). The percentage of cells that show CRD^{hi} scores by cell type, along with the p-value of the corresponding Binomial proportion test, is used for this study. The mean normalized expression of the core circadian genes by the cell type (malignant and non-malignant) is presented in this study. The mean expression of these genes, along with the standard errors of the mean (SEM) for each cell type (malignant and non-malignant cells), are presented in Figure 5. To evaluate differences in gene expression between malignant and non-malignant cells, we conducted a gene-wise Mann–Whitney U test[7]. Statistical significance was annotated as follows. Genes with p-values between 0.01 and 0.05 are marked with '*', those with p-values between 0.001 and 0.01 are denoted by '**', and genes with p-values below 0.001 are indicated by '***'. Genes exhibiting no statistically significant differential expression are labeled as 'n.s' (not significant).

E. 4T1 cell mouse TNBC model:

The 4T1 cells were maintained in RPMI-1640 (ATCC, 30-2001) supplemented with 10% FBS and 1% penicillin and streptomycin in 5% CO_2. Briefly, 10,000 cells suspended in sterile PBS were injected subcutaneously into the 4th mammary gland fat pad. The mice were housed in LD or CRD conditions for 3-4 weeks before they were sacrificed to harvest the tumors. To investigate the effect of immunotherapy on tumor progression and metastasis, BALB/cJ mice were housed under LD 12:12 conditions following injection of 4T1 cells until palpable tumors developed. They were segregated

and assigned randomly either -to LD or CRD conditions. On days 6, 9, and 12 (after the palpable tumors developed), an anti-LILRB4 antibody (ABclonal, #A7073) (polyclonal, 50μg) was injected intratumorally. The animals were checked regularly to observe their body weight and tumor sizes.

F. Histology and tumor burden analysis:

The tumors and the mammary glands were harvested using aseptic techniques. Tumor volumes were calculated for each breast tumor using caliper measurements after every 3 days. $Tumor\ volume\ = (length\ \times width\hat{}2\ )/2$ was used to measure the volume of the tumors [8], whereas the equation "$Tumor\ burden\ (\%)\ = (Tumor\ weight\ /\ mice\ body\ weight) \times 100\ \%$" was considered to determine the tumor burden[9]. The tumor, or the lung samples were fixed in 10% neutral buffered formalin (NBF) for 48 hours and were stained with hematoxylin and eosin (H&E) at the Texas A&M Histology Core Facility. The tissue sections were stained with hematoxylin and eosin (H&E) for pathological examinations.

G. Blood chemistry and cytokine array:

Pre-mortem terminal blood was collected from the FVB-Tg(C3-1-Tag)cJeg/JegJ mice. The blood samples were transferred to the Texas A&M TVMDL to analyze the blood chemistry parameters. The cytokine array was performed using the Proteome Profiler Mouse XL Cytokine Array kit (R&D Systems, ARY028). The blood samples were kept undisturbed for 30 minutes at room temperature before being spun at 2000 x g for 10 minutes at 4°C. The serum was separated for the cytokine array profiling according to the manufacturer's protocol and were scanned using ChemiDoc Imaging System (BioRad, 12003153).

H. Multiplexing Immunostaining (MxIF Staining) of Mouse Tissues: The panels were developed and optimized for use in mouse tissue. The following primary antibodies

from Fortis Life Sciences, Cell Signaling Technology, and ABclonal were utilized for both immunohistochemical and immunofluorescence staining: rabbit anti-mouse ARG1 [BLR161J], rabbit anti-human/mouse LILRB4 [A7073], rabbit anti-human/mouse CD163 [BLR087G], rabbit anti-mouse CD8a [BLR173J], and rabbit anti-mouse FOXP3 [D6O8R]. Antibody concentration and staining order were optimized using FFPE mouse immune tissue microarray serial sections. Each target was evaluated for overall signal: noise ratio, loss of signal intensity, and overall autofluorescence in the first, third, or sixth positions via heat-induced epitope retrieval (HIER) methods. The final optimized order was then subjected to the Opal method of multiplex immunofluorescence (see below). Multiplex immunofluorescence staining was performed following the protocol mentioned by Lei *et al.*[10] with the Akoya Opal™ Polaris 7-color IHC kit fluorophores (Akoya Biosciences [NEL861001KT]). FFPE tissue sections were baked for 30 minutes, deparaffinized in xylene, and rehydrated by serial passage through graded concentrations of ethanol. Endogenous peroxidase in tissues was blocked with 0.9% H_2O_2/methanol for 40 min. Multiple (7) sequential HIER treatments were performed for 20 min each at 92-96°C in Citrate pH 6.0 (first cycle) or Tris EDTA pH 9 (remaining cycles) buffer. After each HIER cycle, sections were rinsed with DI water and cooled at RT for 20 minutes. Tissue sections were circled with hydrophobic barrier pens and blocked with 20% normal goat serum for 20 minutes before incubation with primary antibody for 20 min. Then, sections were rinsed with TBST for 10 min and incubated with HRP-conjugated secondary (A120-501P) for 20 min, followed by another 10 min rinse in TBST. Incubation with an Opal fluorophore such as Opal480 (cyan), Opal520 (green), Opal570 (yellow), Opal620 (orange), Opal780 (white) was then done for 10 min, followed by a 10 min rinse in DI water. Bound primary and secondary antibodies were then subjected to the next HIER

treatment (as aforementioned) for 20 min. After washing in DI water and cooling at RT for 20 min, the process of staining and antibody removal was repeated for the desired number of targets. Finally, after staining with the sixth Opal fluorophore (TSA-DIG), an additional HIER cycle (7th) was performed. Finally, the tissue specimens were stained with 4′,6-diamidino-2-phenylindole (DAPI) for 10 min and mounted in VECTASHIELD Vibrance Antifade Mounting Medium (ThermoFisher Scientific). Akoya Biosciences' PhenoImager HT (formerly known as Vectra Polaris Automated Quantitative Pathology Imaging System) was used for multispectral imaging at 40× magnification.

I. Tumorsphere and organoid assay:

The single cells (from tumors) were cultured in ultra-low attachment 96-well plates in EpiCult™-B (mouse) medium. 500 cells per well from LD and CRD group tumors were added and incubated under standard cell culture conditions for 5 days in the CO_2 incubator before being observed under the microscope. For the organoid assay, singlet cells were resuspended in Geltrex™ and plated on a 24-well plate. EpiCult™-B medium was added to grow the organoids. After 7 days of incubation, the organoids formed were imaged under the microscope.

J. Western blot analysis:

Western blot analysis was performed using a standard protocol with minor modifications [11, 12]. Briefly, proteins from tumor tissues were used for SDS-PAGE and subsequently transferred onto PVDF membranes. The membranes were blocked with blocking buffer (CAS-Block™, Invitrogen) and were incubated with specific primary antibodies, followed by horseradish peroxidase (HRP)-conjugated secondary antibodies. Detection was performed using the SuperSignal™ West Pico PLUS

Chemiluminescent Substrate (Thermo Fisher Scientific). Band intensity was also quantified by ImageJ software.

K. Statistical analysis:

GraphPad Prism8 was used as the Statistical analysis tool, except for the assessment of the rhythmicity, which was carried out using the JTK cycle. Each experiment was independently repeated three times. The exact sample size (n) in each group for survival analysis is provided in the figures. The significance was assessed using either an Unpaired t-test or analysis of variance (ANOVA), with a false positive threshold of 0.05 considered acceptable ($p<0.05$). Unless otherwise mentioned, the data were presented as mean ± standard error of the mean (SEM).

Supplementary Figures:

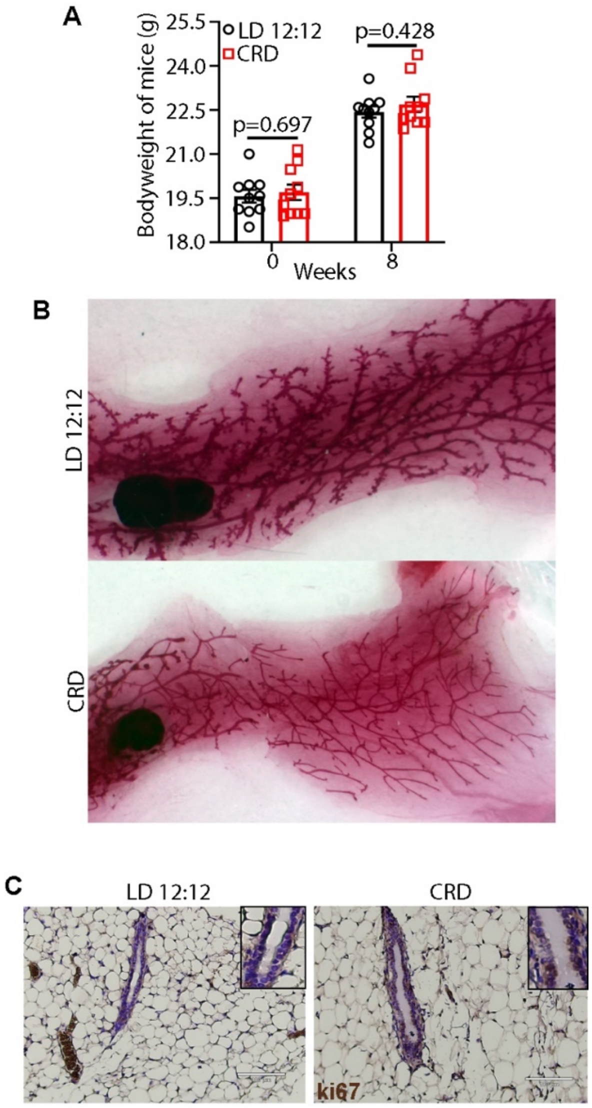

Figure S1. The effect of CRD on mammary glands. **(A)** To investigate the effect of CRD on body weight, the body weight of the LD and CRD-induced mice were measured at week 0 and week 8 (n= 10). **(B)** The whole mount images (viewed under 4X) of LD and CRD-induced mammary glands are shown (n= 3). **(C)** Staining to identify Ki67-positive cells in the mammary glands of both LD and CRD groups. Scale bar: 100 µm. *p* values represent the significance level from an unpaired t-test.

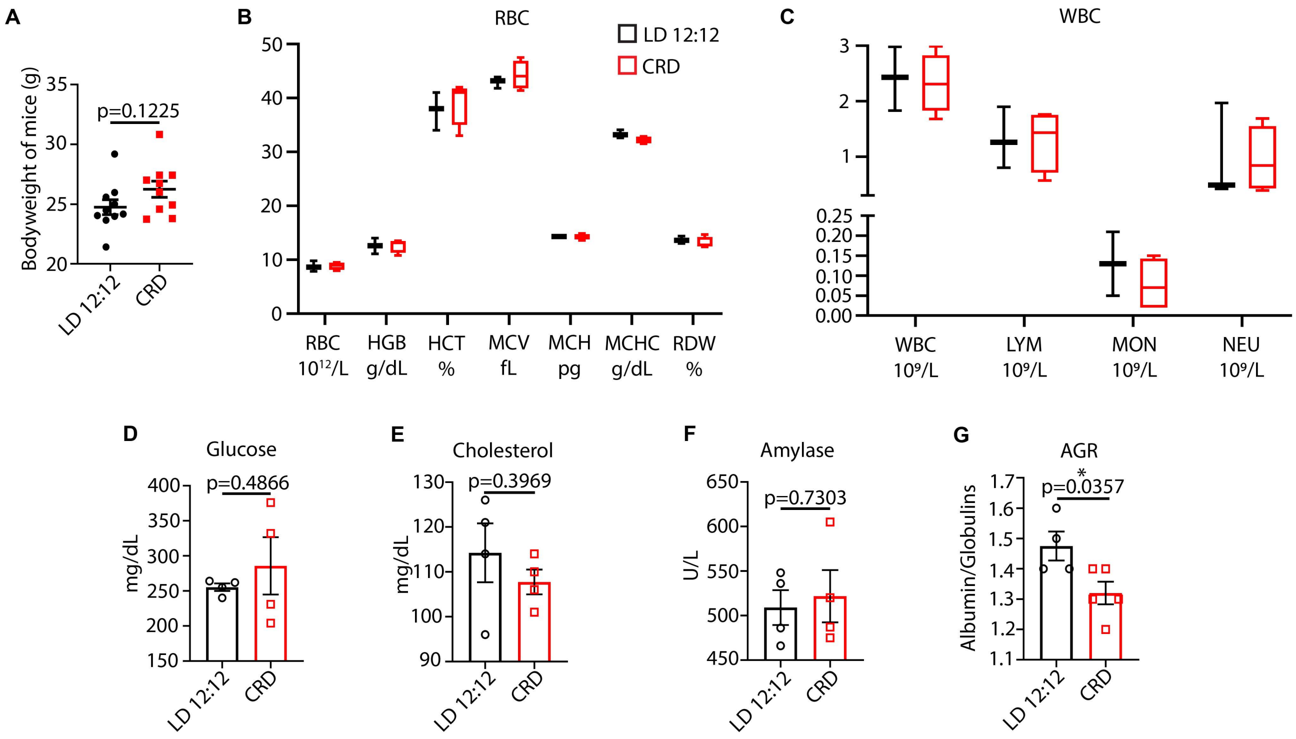

Figure S2. CRD does not alter blood cell counts. Female (FVB-Tg(C3-1-TAg)cJeg (C3-TAg) mice were housed in LD 12:12 or CRD conditions, and the weights were recorded at the end of the experiment (n=10) **(A).** The total numbers of red blood cells (RBC) **(B)** and white blood cells (WBC) **(C)** in LD (n =3) and CRD (n =4) mice were counted. HGB, hemoglobin; HCT, hematocrit; MCV, mean corpuscular volume; MCH, mean corpuscular hemoglobin; MCHC, mean corpuscular hemoglobin concentration; RDW, red cell distribution width; LYM, lymphocytes; MON, monocytes; NEU, neutrophils. Data presented as box-and-whisker plots. Variability is shown using medians (line in the box), 25th and 75th percentiles (box), and min to max (whiskers). The blood profile of some metabolites such as glucose (n=4 LD and n=4 CRD) **(D),** total cholesterol (n=4 LD and n=4 CRD) **(E),** amylase (n=4 LD and n=4 CRD) **(F),** and albumin to globulin ratio (AGR) (n = 4 LD and n = 5, CRD) **(G)** in LD and CRD-induced mice. p values represent the significance level from an unpaired t-test where $*p < 0.05$ indicates a statistically significant difference between the groups.

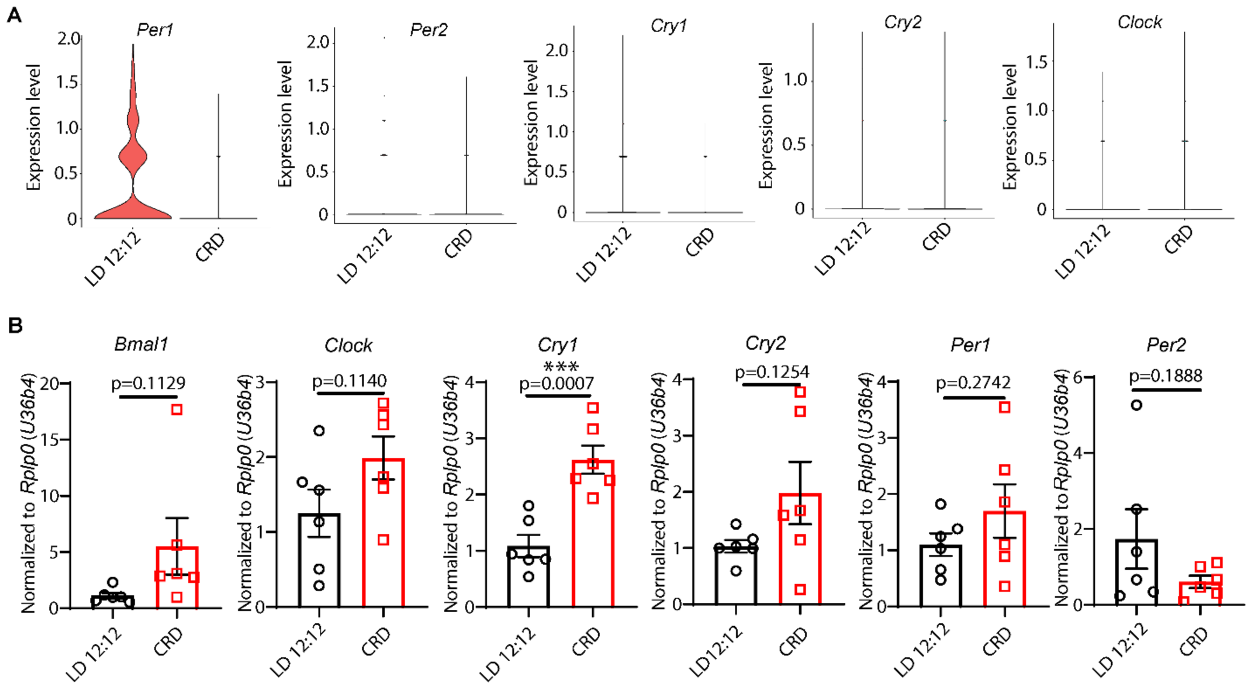

Fig. S3. Core clock gene expression in tumors. Expression of clock genes (*Per1, Per2, Cry1, Cry2, Bmal1, Clock*) in response to circadian rhythm disruption (CRD) is shown using scRNA-seq **(A)** and real-time PCR (n=6) **(B).** Gene expression was normalized to *Rplp0 (U36b4)*. *p*-values represent the significance level from an unpaired t-test where $*p < 0.05$ indicates a statistically significant difference between the groups.

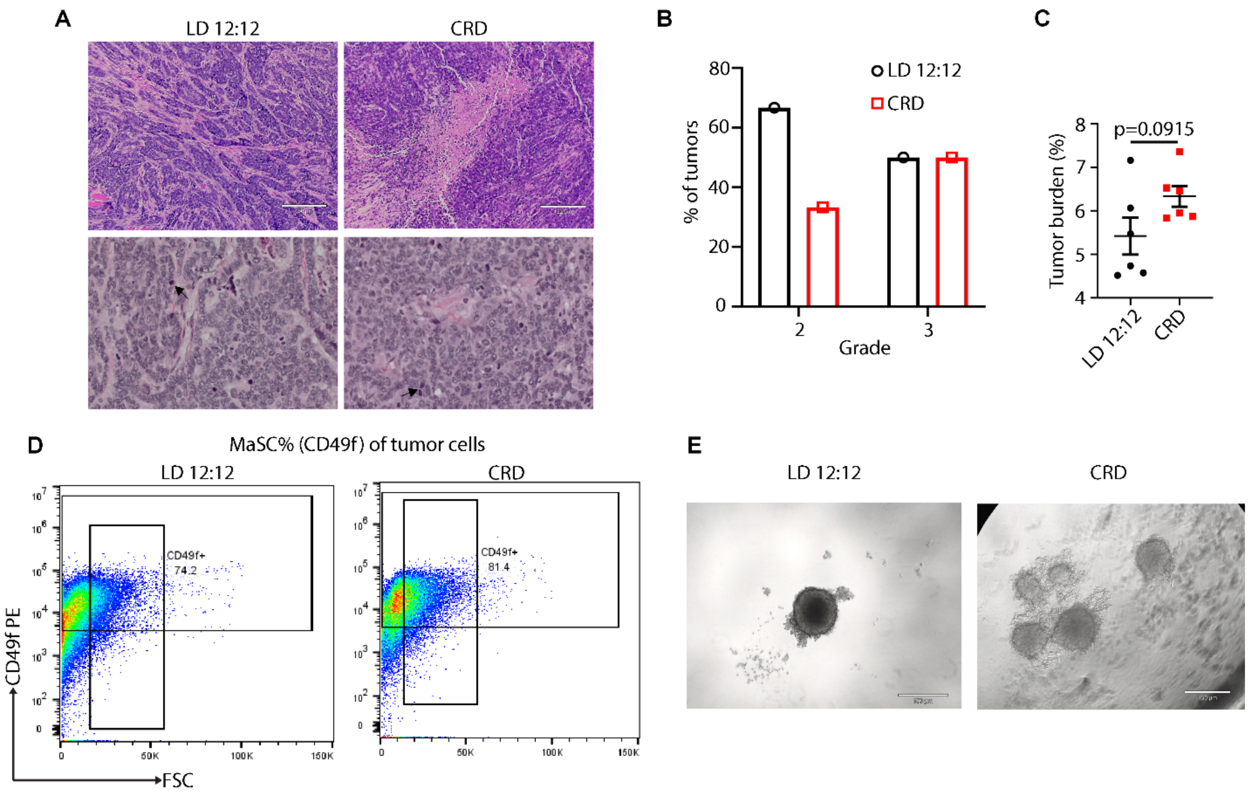

Fig. S4. **Histological analysis of LD and CRD-induced tumors.** H&E-stained tumors are shown **(A),** scale bar=150 µm, and were analyzed and graded according to previous reports [13]. The data are presented as a bar graph **(B)** (n=4 for CRD, n=3 for LD). The graphical representation of tumor burden in LD and CRD-induced 4T1 tumors (n=6) **(C). (D)** CD49f marker profile prepared using flow cytometry. The mammosphere forming efficiency of LD and CRD tumor cells is shown in **E.** Scale bar: 150 µm. *p* values represent the significance level from an unpaired t-test.

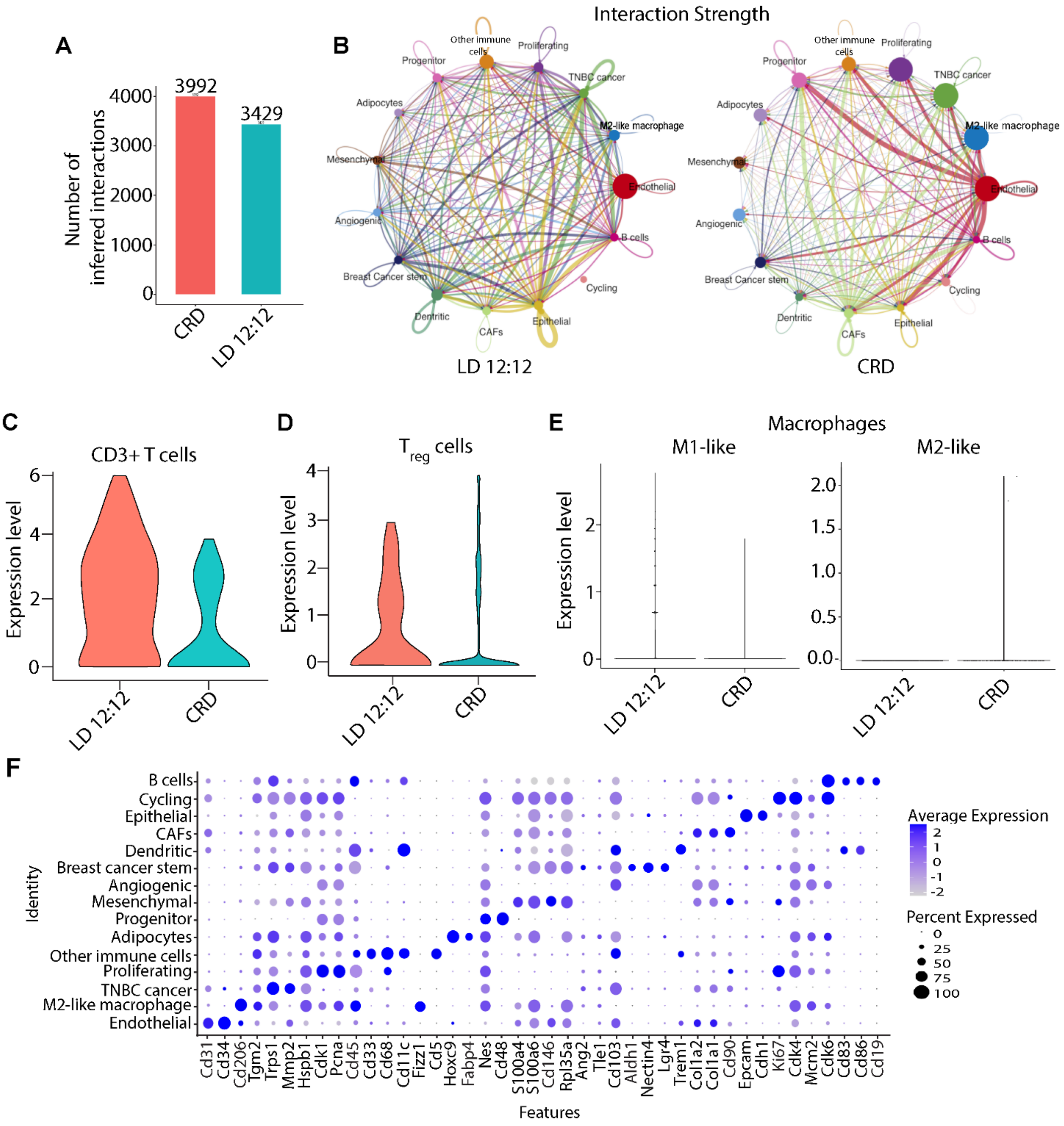

Fig. S5. CRD alters the cell-cell interactions in the tumor microenvironment. CellChat analysis revealed 3429 and 3992 significant ligand-receptor interactions between cells in LD and CRD tumors, respectively **(A).** The interaction strength has been shown in **B.** Violin plots using scRNA seq data showing that the expression of CD3^{+}T cells is reduced in CRD-induced tumors, indicating a decrease in immune response **(C),** whereas increased expression levels of regulatory T (T$_{reg}$) cells are observed under CRD conditions **(D)**. Expression levels of M1-like macrophage populations are low and M2-like macrophage populations are high in CRD-induced tumors compared to LD tumors **(E)**. **(F)** Dot plot depicting the top-expressed genes (markers) across different cell populations in the tumor microenvironment.

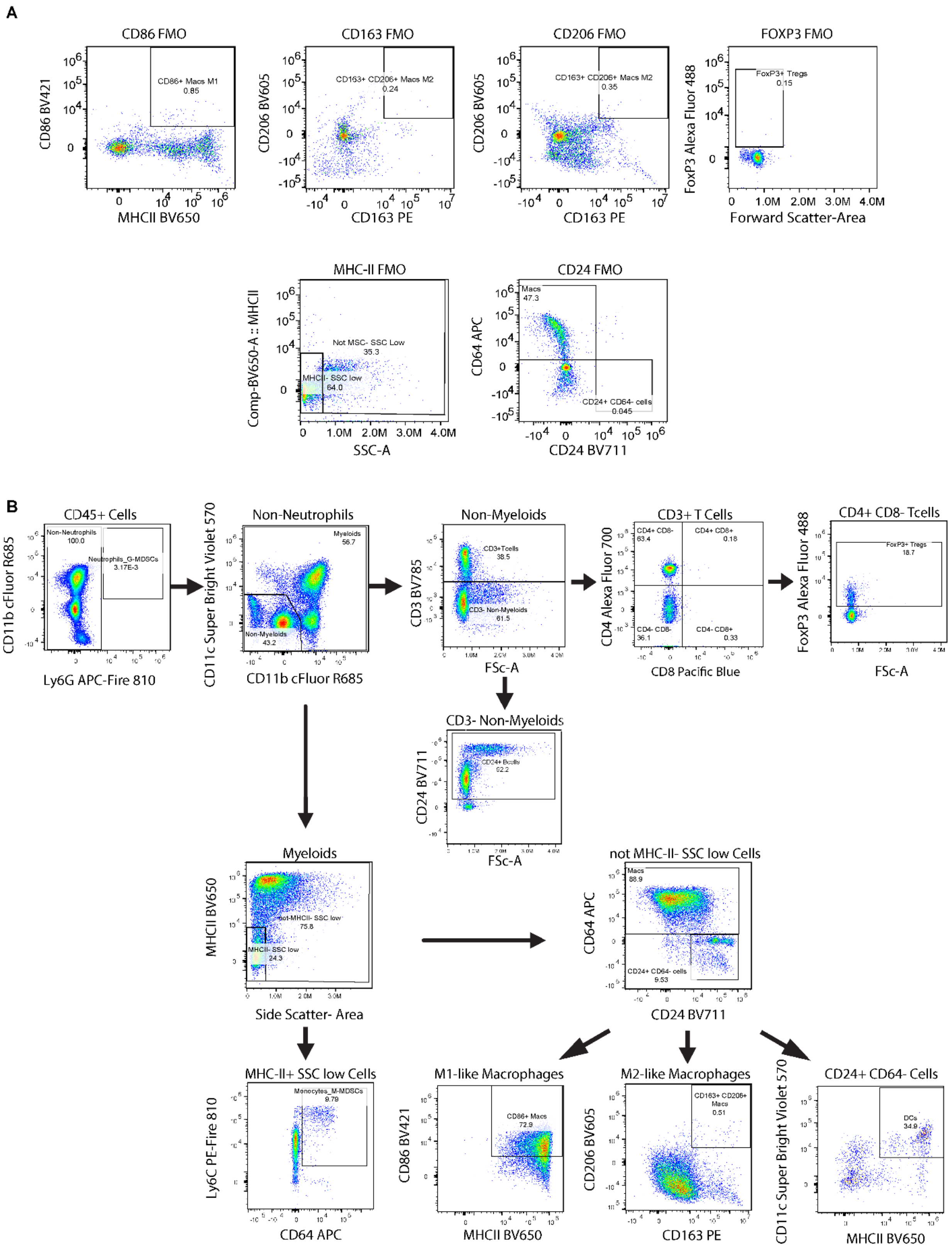
A
CD86 FMO
CD86+ Macs M1
0.85
CD86 BV421
MHCII BV650
CD163 FMO
CD163+ CD206+ Macs M2
0.24
CD206 BV605
CD163 PE
CD206 FMO
CD163+ CD206+ Macs M2
0.35
CD206 BV605
CD163 PE
FOXP3 FMO
FoxP3+ Tregs
0.15
FoxP3 Alexa Fluor 488
Forward Scatter-Area
MHC-II FMO
Not MSC- SSC Low
35.3
MHCII- SSC low
64.0
Comp-BV650-A :: MHCII
SSC-A
CD24 FMO
Macs
47.3
CD24+ CD64- cells
0.045
CD64 APC
CD24 BV711
B
CD45+ Cells
Non-Neutrophils
100.0
Neutrophils_G-MDSCs
3.17E-3
CD11b cFluor R685
Ly6G APC-Fire 810
Non-Neutrophils
Myeloids
56.7
Non-Myeloids
43.2
CD11c Super Bright Violet 570
CD11b cFluor R685
Non-Myeloids
CD3+Tcells
38.5
CD3- Non-Myeloids
61.5
CD3 BV785
FSc-A
CD3+ T Cells
CD4+ CD8-
63.4
CD4+ CD8+
0.18
CD4- CD8-
36.1
CD4- CD8+
0.33
CD4 Alexa Fluor 700
CD8 Pacific Blue
CD4+ CD8- Tcells
FoxP3+ Tregs
18.7
FoxP3 Alexa Fluor 488
FSc-A
CD3- Non-Myeloids
CD24+ Bcells
62.2
CD24 BV711
FSc-A
Myeloids
not-MHCII- SSC low
75.8
MHCII- SSC low
24.3
MHCII BV650
Side Scatter- Area
not MHC-II- SSC low Cells
Macs
88.9
CD24+ CD64- cells
9.53
CD64 APC
CD24 BV711
MHC-II+ SSC low Cells
Monocytes_M-MDSCs
9.79
Ly6C PE-Fire 810
CD64 APC
M1-like Macrophages
CD86+ Macs
72.9
CD86 BV421
MHCII BV650
M2-like Macrophages
CD163+ CD206+ Macs
0.51
CD206 BV605
CD163 PE
CD24+ CD64- Cells
DCs
34.9
CD11c Super Bright Violet 570
MHCII BV650

Fig. S6. Flow cytometry Fluorescence Minus One (FMO) and gating strategy. The FMO controls are shown in **(A).** The gating strategy is used to identify CD45, macrophages (M1-like and M2-like), T-cells, T_{reg} (FOXP3), MDSCs, and dendritic cells (n=4).

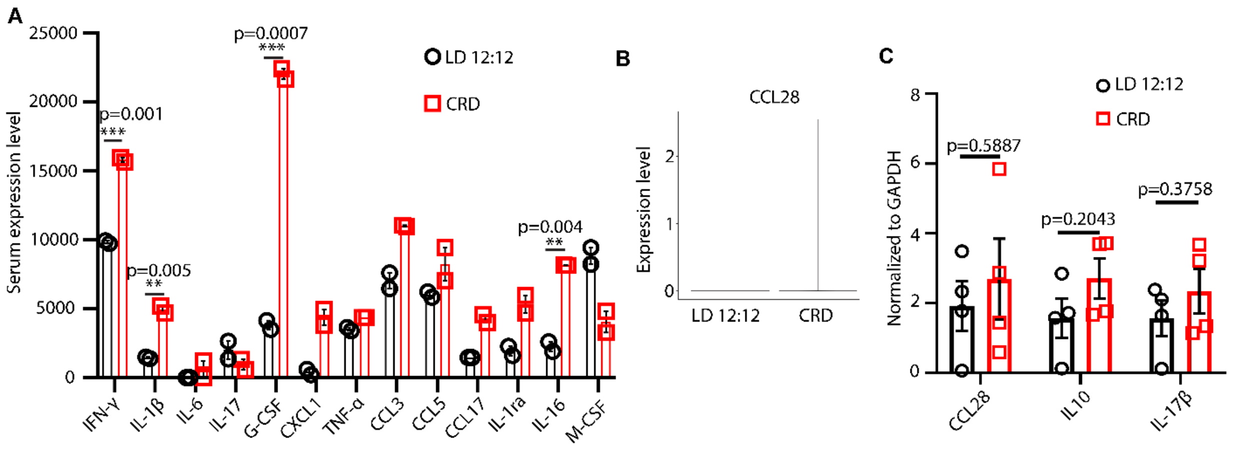

Fig. S7. Cytokine and chemokine profile in LD and CRD mice. (A) Proteome Profiler Mouse XL Cytokine Array kit was used to determine the circulating cytokine/chemokine concentrations in the CRD and LD mice. n=2 independent preparations for each condition **(A).** Quantitative estimation was performed by normalizing the densitometry value for each sample to that of a control. **(B)** CRD enhances *Ccl28* expression in mice, as shown by scRNA seq. **(C)** The transcript levels of *Ccl28, Il10*, and *Il-17β* in LD and CRD-induced tumors were analyzed using real-time PCR (n=4). *p*-values represent the significance level from an unpaired t-test where $^{**}p < 0.01$, $^{***}p < 0.001$ indicates a statistically significant difference between the groups.

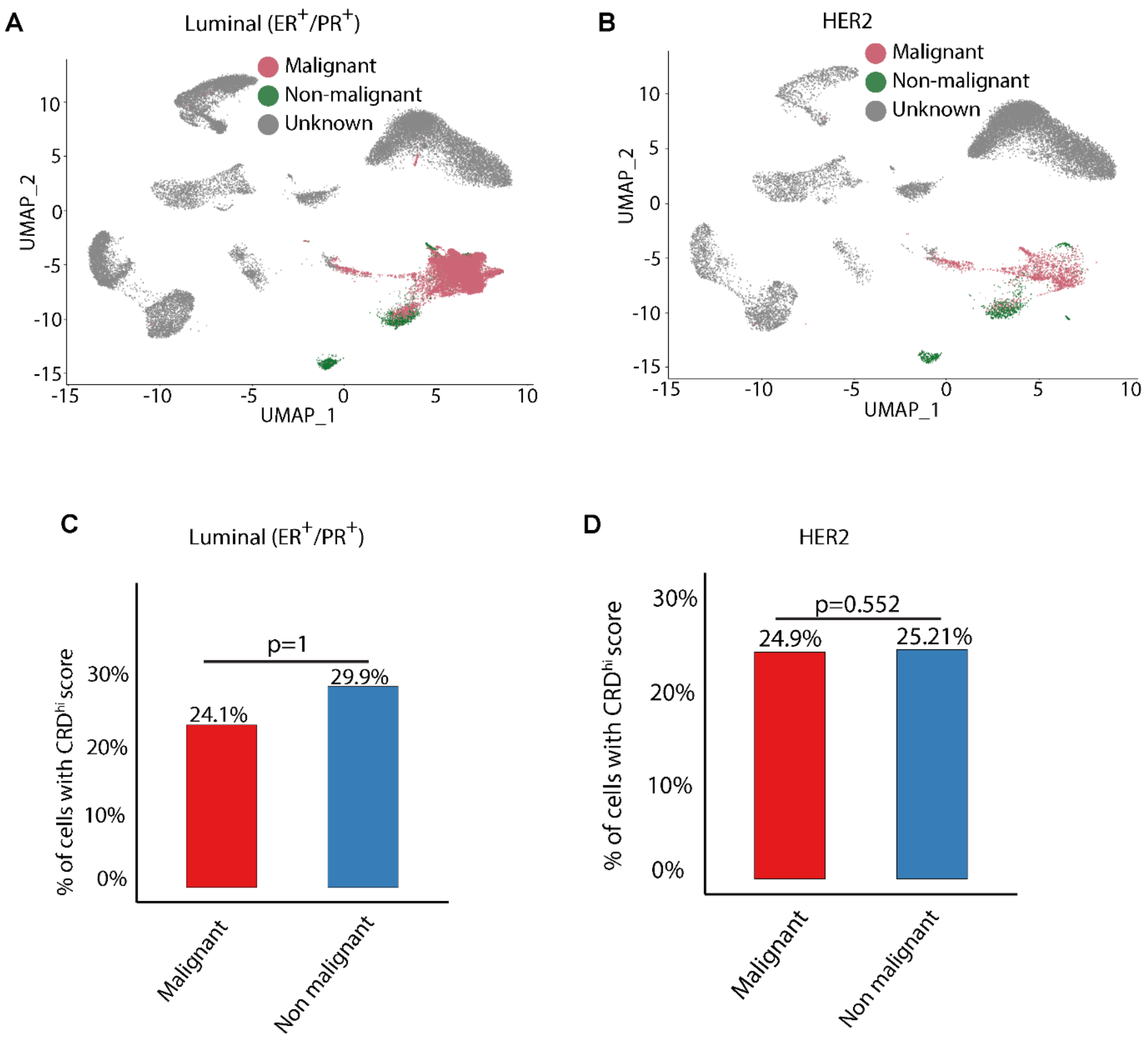

Fig. S8. Circadian rhythm is disrupted in the malignant cells of the aggressive tumors. UMAP embeddings corresponding to luminal **(A)** and HER2^{+} **(B)** patients only after data harmonization colored by inferCNV cell types. These single cells with known inferCNV calls (14,308 cells combined across 9 patients for luminal and 2,743 cells combined across 4 patients with HER2^{+}) were used for downstream analysis. The percentage of malignant and non-malignant cells with CRD^{high} in ER^{+} /PR^{+}tumor **(C)** and HER2^{+} tumor **(D)** is shown. The percentage of cells that show CRD^{hi} scores by cell type, along with the *p*-value of the corresponding Binomial proportion test, is used for this study.

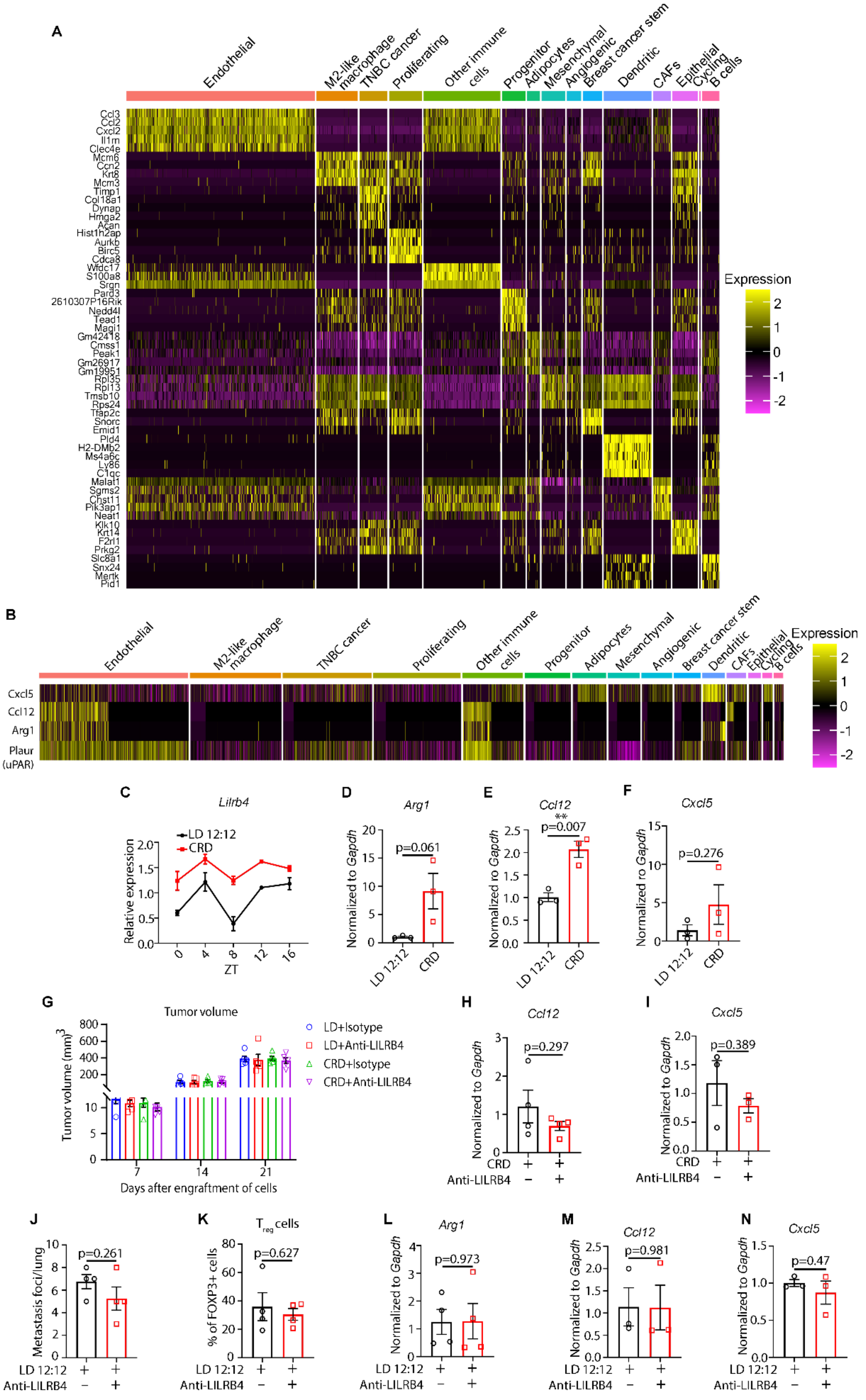

Fig. S9. CRD induces mammary tumorigenesis by enhancing LILRB4 signaling pathway
(A) Heat map of differentially expressed genes in LD tumors using DESeq2 analyses of

scRNA-seq data. **(B)** The DGE of the *Lilrb4*-associated factors (*Arg1, Cxcl4, uPar (Plaur), Ccl12*) from the scRNA seq analysis of CRD-induced tumor. **(C)** Rhythmicity of the LILRB4 in the mammary gland was determined by collecting mammary glands at the indicated time points from LD or CRD-induced (for 8 weeks) mice using JTK_Cycle analyses; *pJTKcycle < 0.05, **pJTKcycle < 0.01, ***pJTKcycle < 0.001, and ****pJTKcycle < 0.0001. **(D)** *Arg1* transcript (target of LILRB4) level was determined in LD and CRD-induced mammary glands using real-time PCR (n=3). The transcript levels of *Ccl12* **(E)** and *Cxcl5* **(F)** were determined using real-time PCR (n=3). Tumor volumes were observed to be invariable across the treatment groups **(G).** The transcript levels of *Ccl12* **(H),** and *Cxcl5* **(I)** were determined using real-time PCR in the LILRB4-targeted immunotherapy in CRD-induced tumors (n=3 or 4). The number of metastatic foci in the lungs of LD (control) and LD (LILRB4-antibody) mice at the time of their sacrifice is shown in **J** (n=4). The percentage of T_{reg} cells in control and LILRB4-antibody-treated LD-induced tumors is shown in **K** (n=4)**.** The transcript level of ARG1 in control and LILRB4-antibody-treated LD-induced tumors is shown by real-time PCR **(L)** (n=4)**.** The transcript levels of *Ccl12* **(M),** and *Cxcl5* **(N)** were determined using real-time PCR in the LILRB4-targeted immunotherapy in LD-induced tumors (n=4). p-values represent the significance level from an unpaired t-test where **$p < 0.01$ indicates a statistically significant difference between the groups.

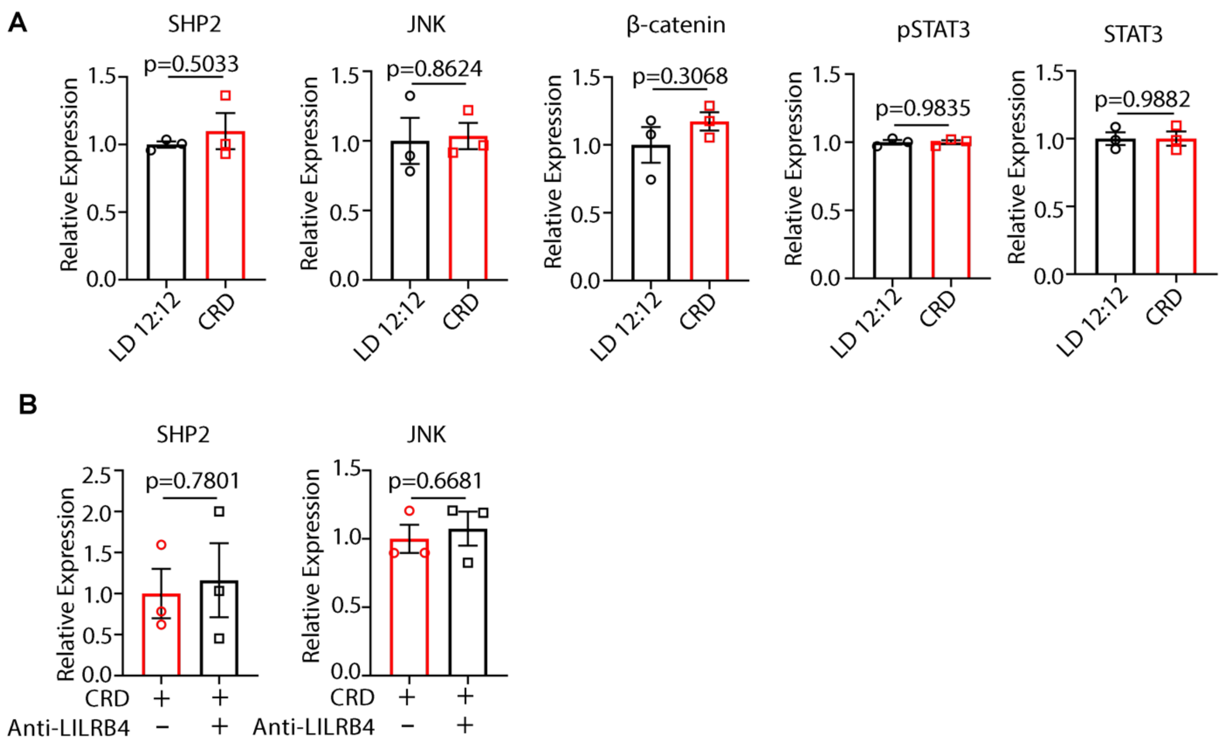

Fig. S10. The densitometric analyses comparing the protein expressions. (A) The densitometric analyses comparing the phospho STAT3 (pSTAT3), total STAT3, β-catenin, JNK and SHP2 protein expressions relative to β-actin in LD and CRD-induced tumor. **(B)** The expression of total JNK and SHP2 following the anti-LILRB4 antibody was represented as relative expression to β-actin. *$p < 0.05$, **$p < 0.01$, ***$p < 0.001$, ****$p < 0.0001$ represents the significance level from an unpaired t-test.

Supplementary Data

Data S2: List of Primers

Genes	Strand	Sequence
Rplp0 (U36b4)	Forward	5’- AGATTCGGGATATGCTGTTGGC -3’
	Reverse	5’- TCGGGTCCTAGACCAGTGTTC -3’
Gapdh	Forward	5’- AAC AGC AAC TCC CAC TCT TC -3’
	Reverse	5’- CCT GTT GCT GTA GCC GTA TT -3’
Clock	Forward	5’- CCA CCA CAG CAG TTC TTA CA -3’
	Reverse	5’- TGC TCT GTT GTA GTG GAA AGG -3’
Cry1	Forward	5’- CTG AGG CAA GCA GAC TGA ATA -3’
	Reverse	5’- CCC TCC ATT CCC ATT AGA GTT AG -3’
Cry2	Forward	5’- AGA GAC TAG TCG GCT CAA CA -3’
	Reverse	5’- GGA AGG GAC AGA TGC CAA TAG -3’
Per1	Forward	5’- CAG TCA GAG CAG CCA TAC AA -3’
	Reverse	5’- GTC CTG GAG CAC ACA CTT AAT -3’
Per2	Forward	5’- AAG CTG TCA CCA CCA TAG AAA G -3’
	Reverse	5’- GCA AGG AGG CTG GTT CTT ATA G -3’
Bmal1	Forward	5’- CAT CAA GAC GAC ATA GGA CAC C -3’
	Reverse	5’- CAT CGA CTT CGT AGC GTG ATA A -3’
Arg1	Forward	5’- CAT TGG CTT GCG AGA CGT AGA C -3’
	Reverse	5’- GCT GAA GGT CTC TTC CAT CAC C -3’
gp49B (*Lilrb4a*)	Forward	5’- ATT CTG GAA CCC AGG ACT GC -3’
	Reverse	5’- GGA ACC CTG ACA CCA GGT AA -3’
Cxcl5	Forward	5’- CCC TTC CTC AGT CAT AGC CG -3’
	Reverse	5’- CTA TGA CTT CCA CCG TAG GGC -3’
Ccl12	Forward	5’- GCT ACC ACC ATC AGT CCT CAG -3’
	Reverse	5’- GAC ACT GGC TGC TTG TGA TTC -3’
Ccl28	Forward	5’- CAA GCA GGG CTC ACA CTC AT -3’

	Reverse	5’- GGC CAT GGG AAG TAT GGC TT -3’
Il17b	Forward	5’- GGA CTG GCC GCA CAG C -3’
	Reverse	5’- GCC TCC CTT GCC CTT TTC TT -3’
Il10	Forward	5’- AGG CGC TGT CAT CGA TTT CT -3’
	Reverse	5’- ATG GCC TTG TAG ACA CCT TGG -3’

Data S3: List of Antibodies

**Target**	**Isotype**	**Conjugate**	**Clone**	**Company**	**Cat #**
ki67	Rabbit IgG	Unconjugated	-	Abclonal	A16919
αSMA	Rabbit IgG	Unconjugated	-	Abclonal	A1011
CD4	Rat IgG2b, κ	Alexa Fluor® 700	RM4-4	BioLegend	116022

CD8a	Rat IgG2a, κ	Pacific Blue™	53-6.7	BioLegend	100725
CD86	Rat IgG2a, κ	BV421	PO3	BioLegend	105123
CD163	Rat IgG2a, κ	PE	S15049I	BioLegend	155308
CD45	Rat IgG2b, κ	Alexa Fluor® 594	30-F11	BioLegend	103144
FOXP3	Mouse IgG1, κ	Alexa Fluor® 488	150D	BioLegend	320011
CD64	Mouse IgG1, κ	APC	X54-5/7.1	BioLegend	139306
Ly-6G	Rat IgG2a, κ	APC-Fire 810	1A8	BioLegend	127670
Ly-6C	Rat IgG2c, κ	PE-Fire 810	HK1.4	BioLegend	128061
MHC Class II (I-A + I-E)	Rat IgG2b, κ	BV650	M5/114.15.2	Thermo Fisher Scientific	416-5321-82
CD11c	IgG	SBV570	N418	Bio-Rad	MCA1369SBV570
CD206	Rat IgG2a, κ	BV605	C068C2	BioLegend	141721
CD24	Rat IgG2b, κ	BV711	M1/69	BioLegend	101851

CD3	Rat IgG2b, κ	BV785	17A2	BioLegend	100231
CD11b	Rat IgG2b, κ	cFluor R685	M1/70	Cytek Biosciences	R7-20547
Viability	-	Zombie NIR	-	BioLegend	423105
CD16/32	Rat IgG2a, λ	-	93	BioLegend	101302
LILRB4	Rabbit IgG	Unconjugated	-	Abclonal	A7073
pSHP2	Rabbit IgG	Unconjugated	-	Abclonal	AP0267
SHP2	Rabbit IgG	Unconjugated	-	Abclonal	A2793
pJNK	Rabbit IgG	Unconjugated	-	Abclonal	AP1163
JNK	Rabbit IgG	Unconjugated	-	Abclonal	A18678
β-catenin	Rabbit IgG	Unconjugated	-	Abclonal	A19657
pSTAT3	Rabbit IgG	Unconjugated	-	Abclonal	AP0474
STAT3	Rabbit IgG	Unconjugated	-	Abclonal	A1192
c-FOS	Rabbit IgG	Unconjugated	-	Abclonal	A0236
c-MAF	Rabbit IgG	Unconjugated	-	Abclonal	A12720

ARG1	Rabbit IgG	Unconjugated	-	Abclonal	A1847
β-actin	Mouse IgG	HRP-conjugated	-	Cell Signaling Technology	12262S
Anti-Rabbit	Goat IgG	HRP-conjugated	-	Cayman chemical	10004301
Anti-Mouse	Goat IgG	HRP-conjugated	-	Cayman chemical	10004302
Anti-Rabbit	Rabbit IgG	Alexa Fluor® 488	-	Cell Signaling Technology	4412S
IHC Kit	Mouse and Rabbit	HRP-conjugated	-	Abcam	ab64264

References:

1 Finak G, McDavid A, Yajima M, Deng J, Gersuk V, Shalek AK *et al.* MAST: a flexible statistical framework for assessing transcriptional changes and characterizing heterogeneity in single-cell RNA sequencing data. *Genome Biology* 2015; 16: 278.

2 Wu T, Hu E, Xu S, Chen M, Guo P, Dai Z *et al.* clusterProfiler 4.0: A universal enrichment tool for interpreting omics data. *The Innovation* 2021; 2: 100141.

3 Wu SZ, Al-Eryani G, Roden DL, Junankar S, Harvey K, Andersson A *et al.* A single-cell and spatially resolved atlas of human breast cancers. *Nat Genet* 2021; 53: 1334-1347.

4 Hao Y, Hao S, Andersen-Nissen E, Mauck WM, Zheng S, Butler A *et al.* Integrated analysis of multimodal single-cell data. *Cell* 2021; 184: 3573-3587.e3529.

5 Korsunsky I, Millard N, Fan J, Slowikowski K, Zhang F, Wei K *et al.* Fast, sensitive and accurate integration of single-cell data with Harmony. *Nat Methods* 2019; 16: 1289-1296.

6 He L, Fan Y, Zhang Y, Tu T, Zhang Q, Yuan F *et al.* Single-cell transcriptomic analysis reveals circadian rhythm disruption associated with poor prognosis and drug-resistance in lung adenocarcinoma. *J Pineal Res* 2022; 73: e12803.

7 Mann HB, & Whitney, D. R. . On a test of whether one of two random variables is stochastically larger than the other. *The Annals of Mathematical Statistics* 1947; 18: 50-60.

8 Kersemans V, Cornelissen B, Allen PD, Beech JS, Smart SC. Subcutaneous tumor volume measurement in the awake, manually restrained mouse using MRI. *Journal of Magnetic Resonance Imaging* 2013; 37: 1499-1504.

9 Hadadi E, Taylor W, Li XM, Aslan Y, Villote M, Riviere J *et al.* Chronic circadian disruption modulates breast cancer stemness and immune microenvironment to drive metastasis in mice. *Nat Commun* 2020; 11: 3193.

10 Lei Y, VanPortfliet JJ, Chen YF, Bryant JD, Li Y, Fails D *et al.* Cooperative sensing of mitochondrial DNA by ZBP1 and cGAS promotes cardiotoxicity. *Cell* 2023; 186: 3013-3032 e3022.

11 Sarkar TR, Battula VL, Werden SJ, Vijay GV, Ramirez-Pena EQ, Taube JH *et al.* GD3 synthase regulates epithelial-mesenchymal transition and metastasis in breast cancer. *Oncogene* 2015; 34: 2958-2967.

12 Kole L, Sarkar M, Deb A, Giri B. Pioglitazone, an anti-diabetic drug requires sustained MAPK activation for its anti-tumor activity in MCF7 breast cancer cells, independent of PPAR-gamma pathway. *Pharmacol Rep* 2016; 68: 144-154.

13 Elston CW, Ellis IO. Pathological prognostic factors in breast cancer. I. The value of histological grade in breast cancer: experience from a large study with long-term follow-up. *Histopathology* 2002; 41: 154-161.
